# How to Attain an Ultralow Interfacial Tension and a Three-Phase Behavior with a Surfactant Formulation for Enhanced Oil Recovery: A Review. Part 2. Performance Improvement Trends from Winsor’s Premise to Currently Proposed Inter- and Intra-Molecular Mixtures

**DOI:** 10.1007/s11743-013-1485-x

**Published:** 2013-05-03

**Authors:** Jean-Louis Salager, Ana M. Forgiarini, Laura Márquez, Lisbeth Manchego, Johnny Bullón

**Affiliations:** Lab. FIRP, Universidad de Los Andes, Mérida, Venezuela

**Keywords:** Enhanced oil recovery, Ultralow tension, Performance improvement

## Abstract

The minimum interfacial tension occurrence along a formulation scan at the so-called optimum formulation is discussed to be related to the interfacial curvature. The attained minimum tension is inversely proportional to the domain size of the bicontinuous microemulsion and to the interfacial layer rigidity, but no accurate prediction is available. The data from a very simple ternary system made of pure products accurately follows the correlation for optimum formulation, and exhibit a linear relationship between the performance index as the logarithm of the minimum tension at optimum, and the formulation variables. This relation is probably too simple when the number of variables is increased as in practical cases. The review of published data for more realistic systems proposed for enhanced oil recovery over the past 30 years indicates a general guidelines following Winsor’s basic studies concerning the surfactant–oil–water interfacial interactions. It is well known that the major performance benefits are achieved by blending amphiphilic species at the interface as intermolecular or intramolecular mixtures, sometimes in extremely complex formulations. The complexity is such that a good knowledge of the possible trends and an experienced practical know-how to avoid trial and error are important for the practitioner in enhanced oil recovery.

## Introduction

In the first part of this review [1] it is shown that the minimum tension in a formulation scan is attained at the so-called optimum formulation, in which the affinity of the amphiphile(s) at interface is exactly the same for the oil and water phases at the given temperature. Hence, a minimum tension occurrence should be sought under such an optimum condition. It was reported that for simple systems containing pure components, the optimum formulation takes place when a simple linear correlation is satisfied by four variables representing the oil, water, and surfactant nature, as well as temperature. At optimum formulation, a minimum tension is attained, but the value of the minimum, which is a measure of the performance for enhanced oil recovery, has not been still clearly related to the formulation.

Some possible trends have been found, but not as the effect of each formulation variable, and with some discrepancies probably due to a very large number of variables in most practical cases. Since this correlation for the attainment of a tension minimum in simple cases systems involves only four variables, it has been thought that the scrutiny of such a simple situation could improve the understanding and that some general tendencies could be found.

Such an analysis is reported in this article based on a published study of very pure systems. It shows for the first time that the tension performance is in effect simply related with the four variables, which are actually the main ones from the physicochemical point of view. It also shows that the characteristics of the iso-performance contours do not involve any minimum in such very simple system, but an improvement in some direction of change until a restriction or limit is attained. As a consequence displacing a limit could be a way to attain a better performance. But when the limit cannot be displaced, another way is necessary to generate a minimum within the feasible range, which is carried out by so-called synergistic effects, which often take place with mixtures of components.

Since Winsor’s premise in the 1950s, many studies have reported the performance of a huge variety of surfactants and co-surfactants, with different head and tail groups, and depending on the other variables like oil (E)ACN, brine salinity, and temperature. Thanks to the clearer understanding of the performance variations through the analysis of the simple system cases, an organized review to the more complex practical system can be proposed, with some ideas for potential future improvement. This is what is presented in this second part of the review, but the techniques to study experimentally the performance improvement through mixtures will be discussed in the third part [2].

## Concepts and Phenomenology Around the Tension Minimum at Optimum Formulation

Improving the understanding by using systems which do not exhibit complex behavior, i.e. pure surfactant, pure oil, and simple brine, provided the basic know-how on the physico-chemical formulation effect on surfactant–oil–water systems.

Most of the very fundamental studies on the way the interfacial tension changes with formulation and passes through a minimum, have been carried out with pure nonionic surfactants of the type of single isomers of *n*-alkylethoxylates named CiEj, pure *n*-alkane and water, where “i” is the number of carbon atoms in the alkyl tail and “j” the number of ethylene oxide groups in the head [3]. In this kind of system, the formulation alteration is produced by a change in temperature, whose rise tends to decrease the hydrophilicity of the polyethyleneoxide head group by dehydration, and thus to result in the WI → WIII → WII phase behavior transition. These fundamental studies have been carried out after empirical studies on commercial surfactants of the anionic type, often with alcohol as cosurfactant, i.e. more real situations with a much larger number of variables. The fundamental studies are, however, discussed here before the practical cases of commercial surfactant systems, because they are simpler to understand and interpret as far as the trends are concerned.

**Fig. 1:**
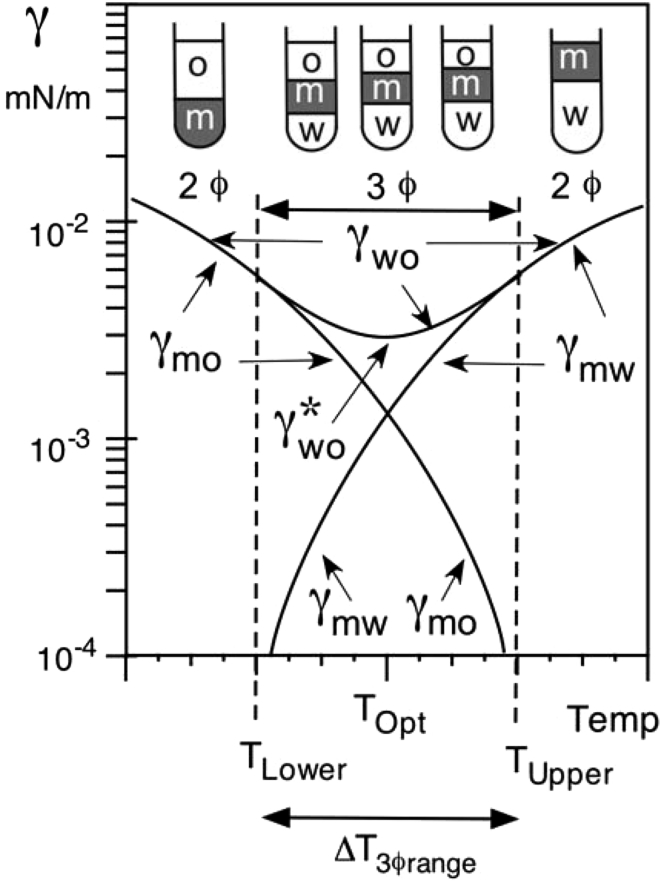
Variations of the interfacial tensions close to optimum formulation, in the case of a nonionic surfactant with a formulation scan produced by a change in temperature

Figure 1 indicates the variation of the interfacial tensions with temperature inside and close to the three-phase zone, which happens in the interval Δ*T*_3ϕrange_ = *T*_Lower_ − *T*_Upper_. The temperature at optimum formulation *T*_opt_ corresponds to Winsor’s *R* = 1 or SAD = 0 situation (see Part 1 [1]), which is essentially located at the center of the three-phase behavior zone. The subscript “m” used for the tensions shown in Fig. 1 indicates the surfactant rich phase, which could be water (at *R* < 1, SAD < 0 or *T* < *T*_Lower_), oil (*R* > 1, SAD > 0 or *T* > *T*_Upper_), or the middle phase bicontinuous microemulsion in the three phase zone (at *R* ≈ 1, SAD ≈ 0 or *T* ≈ *T*_opt_). The subscripts “o” and “w” refer to oil and water, particularly excess oil and excess water in equilibrium with a microemulsion in a three-phase system. γ_OW_ is the interfacial tension between the oil and water phases in all cases, which is also indicated as γ_mW_ and γ_mO_ in the two phases zones. In the center of the three-phase region γ_OW_ is the maximum of (γ_mw_, γ_mo_) but smaller than (γ_mw_ + γ_mo_) because the microemulsion does not wet the oil/water interface [4]. Since (γ_mw_ + γ_mo_) passes through a minimum, then γ_OW_ has to have a minimum as well [5], so-called γ*_OW_, which is the performance criterion for optimum formulation (at *T*_opt_ in Fig. 1).

Figure 1 formulation variable is the temperature, but the following concepts are equivalent with any other formulation variable included in SAD/HLD expression seen in Part 1 of this review [1]. It is obvious from Fig. 1 that if the three-phase zone range diminishes, then γ_OW_ is likely to follow more closely γ_mW_ and γ_mO_ and thus undergoes through a lower minimum. It is worth noting that this is an unavoidable relationship between the tension minimum performance and the range of the three phases zone, as will be discussed later.

Theoretical considerations discussed elsewhere [3, 5–10] indicate that the tension is related to the curvature of the surfactant layer located at the boundary of oil and water domains in the microemulsion structure. This comes from the definition of the tension as the derivative of the free energy with respect to area defined by Helfrich theory [11] on the elasticity of lipid layers. If the free energy is supposed to depend only on the bending energy, and is called curvature free energy, then

1 $$\begin{gathered} \gamma_{\text{OW}} = f_{C}\,=\,\left( {\frac{{\partial F_{C} }}{\partial A}} \right)_{T,V,n} = {\text{curvature free energy per unit area}} \hfill \\ = \kappa_{\text{bd}} \left( {H - H_{0} } \right)^{ 2}\,+\,\kappa_{\text{ss}} K = { 1}/ 2\kappa_{\text{bd}} \left( {C_{ 1} + C_{ 2} - { 2 }H_{0} } \right)^{ 2}\,+\,\kappa_{\text{ss}} C_{ 1} C_{ 2} \hfill \\ \end{gathered} $$

where *C*_1_ and *C*_2_ are the principal (orthogonal) curvatures of the single layer surfactant film at the oil/water boundary, *H* is the actual average curvature (*C*_1_ + *C*_2_)/2 of the film, whereas *H*_0_ is the spontaneous curvature. *K* is the Gaussian curvature (*C*_1_ *C*_2_), κ_bd_ the bending elastic modulus or rigidity of the surfactant layer in a droplet microemulsion, κ_ss_ the so-called saddle-splay deformation rigidity for the bicontinuous structure microemulsion in WIII systems.

Microemulsion issues concerning the interfacial tension and bending effects have been discussed elsewhere, [7, 12] particularly in the bicontinuous structure occurrence [4, 8]. With some assumptions [3], the spontaneous curvature H_0_ can be related to the measurable curvature for the sphere reference in droplet microemulsions, which may be experimentally determined through neutron scattering or other techniques, as an average drop radius *R*, mean domain size ξ, or characteristic length ξκ of a bicontinuous microemulsion [4].

When some area of the surfactant layer in a microemulsion structure is replaced by a same area of a flat interfacial surface, it may be said that the surfactant layer unbends, and that the tension measures the bending energy as follows [3]

2 $$\gamma_{\text{OW}} = { 2 }H^{ 2} \left( {\kappa_{\text{bd}} + \kappa_{\text{ss}} } \right) \, - \kappa_{\text{ss}} C_{1} C_{ 2} $$

This expression renders the variation of the interfacial tension close to optimum formulation where *H* ≈ 0, but the Gaussian curvature (*C*_1_ *C*_2_) is not zero. Consequently close to optimum formulation:

3 $$\gamma_{\text{OW}} = \, - \kappa_{\text{ss}} C_{ 1} C_{ 2} $$

or the equivalent in other models

4 $${\text{as}}\;\gamma_{\text{OW}} = \, \left( { 2\kappa_{\text{bd}} + \kappa_{\text{ss}} } \right)/R^{ 2} $$

where *R* is the maximum radius of the droplets or domains [4]

5 $${\text{or as}}\;\gamma_{\text{OW}} = {\text{ Er}}/ 4\pi R^{ 2} $$

where *R* is a domain radius, and Er is the interfacial rigidity which is essentially proportional to the bending modulus [13, 14].

Different curvature models result in slightly different results concerning the average or mean curvature concept, i.e. 1/*H* = ξ, where ξ is a domain size experimentally measured or estimated. In a pioneering article dealing with bicontinuous microemulsion in which a droplet radius does not exists, ξ was substituted by a mean characteristic length ξκ [15], which indicates the persistence length of the surfactant layer, i.e. the distance over which the layer remains flat [4]. It describes the competition between the bending energy and the thermal energy, and is physically different from the average domain size ξ, but seems to be similar in many cases. In any case ξ_max_ has a limit.

Of course an entropic term in the free energy calculation is likely to be important too, in particular to explain the existence of a disordered microemulsion structure instead of an ordered lamellar liquid crystal one, as it often happens in mixtures [4, 7, 8]. A low tension implies a long persistence length, but not too long because it would produce a periodic structure. Since the characteristic length ξκ exponentially increases with the surfactant layer bending rigidity, there is a maximum critical rigidity above which a liquid crystal is produced instead of a microemulsion [15]. This maximum rigidity is what would result in the ultralow tension performance in enhanced oil recovery. This critical value depends on the interactions in the surfactant layer formulation, and some examples are found to be consistent with the theories [8, 16, 17] even if only some qualitative trends are understood as discussed later.

Nevertheless, in spite of producing a significant change in the tension variation, the introduction of an entropic term in the free energy does not change the phenomenology [3] and is often neglected for the sake of simplicity. This simplification might be appropriate as far as the numerical calculation for general trends are concerned, but it is worth noting that it clearly indicates that complex mixtures introducing disorder are probably a feature improving the tension performance even more.

The purpose of this part is to qualitatively discuss how formulation adjustments are likely to improve the tension performance by increasing the bending rigidity, though not too far. In practice this may be done by increasing the rigidity up to the critical value, and to try to displace the critical value beyond which a liquid crystal would result in the system. In other words some changes are likely to alter the characteristic length and the rigidity in different ways, and thus allows a best compromise to be attained [18, 19].

It is generally accepted that the tension is inversely related with the square of the domain size close to the optimum formulation.

6 $$\gamma_{\text{OW}} \approx \, k \, T/\xi^{ 2} $$

where ξ^−2^ = 1/2 (*C*_1_^2^ + *C*_2_^2^), i.e. an equation that fits both the droplet microemulsion regime and the bicontinuous structure [3–5]. As the formulation tends toward the optimum, the domain size increases by swelling until a maximum is attained and any additional oil or water (or both) separates as one (or two) excess phases.

At optimum formulation, the minimum tension corresponds to the maximum domain size, i.e.:

7 $$\gamma_{ \hbox{min} } = \, - \kappa_{\text{ss}} /\xi_{ \hbox{max} }^{ 2} $$

The average or mean curvature H of an amphiphilic film is found to depend on the formulation distance from the optimum, i.e. in the Fig. 1 case [5].

8 $$H \approx \, A_{1}\;(T-T_{\rm opt})\;{\text{and}}\;H\,=\,A_{2}\;{\text{HLD in general, where}}\;A_{1}\;{\text{and}}\;A_{2}\;{\text{are constant coefficients.}}$$

In another model, the net average curvature *H*_n_ is proportional to HLD, this time expressed as the deviation from the optimum salinity in a log scale or other variables found in the SAD expression [13].

According to previous equations, the tension departure from minimum tension varies proportionally to the square of the deviation from the optimum formulation, i.e. HLD^2^, and may be written as:

9 $$\left( {\gamma_{\text{OW}} /\gamma_{ \hbox{min} } } \right) \, - { 1 } = { 2}\kappa_{\text{ss}} H^{ 2}\; {\text{or }} = {\text{ B}}_{ 1} {\text{HLD}}^{ 2} $$

where B_1_ is a coefficient depending on the rigidity κ_ss_ of the surfactant film in the bicontinuous microemulsion and on the maximum domain size at optimum.

A limited study on about 20 simple systems containing alcohol ethoxylate isomerically pure surfactants and *n*-alkanes [20] showed a general plot of interfacial tension versus temperature. This graph is the same for all systems provided that the tension and temperature are expressed in dimensionless variables introducing two characteristics parameters, i.e. the minimum tension γ_min_ and the three phase zone range of temperature Δ*T*_3ϕrange_ = *T*_Upper_ − *T*_Lower_ [20].

**Fig. 2:**
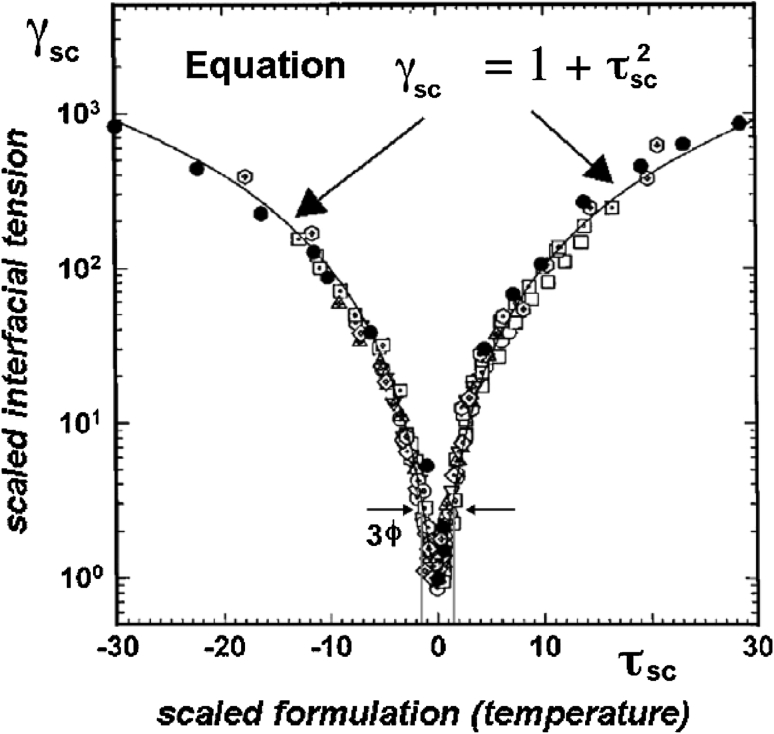
Plot of scaled tension versus scaled temperature with two scaling parameters to characterize the system. Reproduced with authorization from Ref. [20]

Figure 2 plots the scaled tension γ_SC_ versus the scaled temperature τ_SC_, where the minimum tension γ_min_ in the scan is the basic criterion for the optimum formulation performance.

10 $$\gamma_{\text{SC}} = \gamma_{\text{OW}} /\gamma_{ \hbox{min} }\quad {\text{where}}\;\gamma_{ \hbox{min} } = \, - \kappa_{\text{ss}} /\xi_{ \hbox{max} }^{ 2} \,{\text{with}}\;\xi_{ \hbox{max} } = { 2 }/ \, C_{ 1} \left( {T_{\text{Upper}} - T_{\text{Lower}} } \right) $$

where *C*_1_ is a curvature coefficient in the order of 0.001 Å^−1 ^°C^−1^ [5].

It is reported [20] that κ_SS_ is negative and slightly decreases in absolute value as the ξ_max_ increases notably, both tending to decrease the tension minimum with some trend to avoid too much rigidity and liquid crystal formation.

The dimensionless temperature τ and scaled temperature τ_SC _ used are proportional to HLD, as follows

11 $$\tau = { 2 }\left( {T - T_{\text{opt}} } \right)/\left( {T_{\text{Upper}} - T_{\text{Lower}} } \right)\;{\text{ and}}\;\tau_{SC} = \tau \sqrt { - \frac{{2\kappa_{bd} + \kappa_{ss} }}{{\kappa_{ss} }}} $$

All properties and parameters are measured with the exception of the bending rigidity κ_bd_, and the saddle deformation rigidity κ_ss_ of the surfactant monolayer, values which allow us to characterize a system according to the tension minimum and temperature range for three-phase behavior. The significance of these κ parameters, which deal with the elasticity of the surfactant layer, is not easy to relate with the molecular formulation at the interface, but some physical approaches may be found elsewhere [3, 4, 8, 10, 21–26].

It is worth remarking that the minimum tension γ_min_ is low if ξ_max_ is large. This produces a tendency to higher rigidity, but Eq. 10 also states that κ_ss_ should be small to favor the flexibility of the bicontinuous boundary (very close to the flat boundary), which is more stable because of some disorder. It is also seen in Eq. 10 that γ_min_ is lower if the three phase range Δ*T*_3ϕrange_ is narrower since

12 $$\begin{gathered} \gamma_{ \hbox{min}}={\text{ C}}_{ 2} \Updelta T_{{ 3\phi {\text{range}}}}^{2} \,{\text{if }}T{\text{ is the formulation variable}}, \hfill \\ {\text{or in general}}\,\gamma_{ \hbox{min}}={\text{ B}}_{ 3} \Updelta {\text{HLD}}_{{ 3\phi {\text{range}}}}^{2} \,\,{\text{for any formulation variable scanning}}. \hfill \\ \end{gathered} $$

where C2 and B3 are constant coefficients_._

Consequently, it is unlikely that this relationship can be avoided in practice, i.e., a lower tension minimum γ* is to be associated with a narrower 3-phase behavior range, as often reported in the literature where a SAD/HLD parameter variation is often taken into account to measure ΔHLD_3ϕrange_ [27–32].

The generalized plot with the scaling of the tension and the temperature exhibited in Fig. 2 displays an expected correlation according to Eq. 9, i.e., γ_SC_ = 1 + τ_SC_^2^.

The consequence of this simple scaling is that it implies only two independent parameters to characterize the quality of a phenomenology and its performance, which means that the actual number of degrees of freedom to get an optimum formulation is not very large in spite of the fact that there are scores of formulation choices in any practical case. It probably means that many choices are not really independent, and that an actual understanding of what is independent and what is related, and on how to make choices, is probably a top priority for the formulator. The main issue to be analyzed in the optimization is how to get a very high ξ_max_ and a narrow Δ*T*_3ϕrange_ range, but with a relatively small κ_SS_ in absolute values to get some flexibility and avoid the formation of liquid crystals. Unfortunately, there is not enough understanding to predict it yet, and an empirical study of the formulation variables effects on the performance is the only possible step to be carried out.

There is, however, a fairly consistent trend in the data indicating that the three-phase behavior range corresponds to a deviation of τ_SC_ from optimum of about 1.4, and that at the extremes of this range, i.e. at *T*_Upper_ and *T*_Lower_, the γ_OW_ tension is about 3 times higher than its minimum value γ*, as indicated in Fig. 2. In other words, a very small variation of formulation from the optimum results in a considerable increase in the tension from the minimum value. This is why a low tension minimum necessary to warrant a good performance in enhanced oil recovery, is not sufficient. A concomitant accurate control of the formulation during the process is absolutely necessary.

## Relation Between Tension and Solubilization: Performance Index

**Fig. 3:**
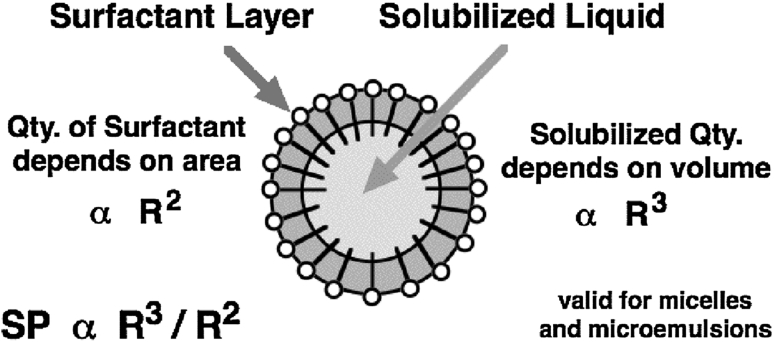
Solubilization increases with the domain size

The curvature of the structure is also known to influence the solubilization in microemulsion. For the sake of simplicity in a qualitative estimation, a spherical shape may be taken as an approximation of the solubilized oil and water domains in a microemulsion, even a bicontinuous one. In such a domain, the amount of oil and water depends on the volume of the domain, i.e. it is *V* = 4/3 π*R* ^3^, where *R* is the radius of the sphere. On the other hand the amount of surfactant that wraps up the domain as indicated in Fig. 3, is proportional to the surface area of the sphere, i.e. *S* = 4π*R* ^2^. Consequently the solubilization parameter (SP), i.e. the volume of oil or water in the microemulsion divided by the amount of surfactant (*V*/*S*) is proportional to the radius *R*. Hence, the general trend is that the larger the structure size ξ, the lower its curvature *H*, and the higher the solubilization, no matter what the exact structure shape is.

A systemic consequence is that the lower the tension, the larger the domain size and the less transparent the microemulsion, because in this range, a larger structure results in increased light scattering. In other words, it means that “good” microemulsions are not transparent, as often found as a microemulsion characteristic in the literature more than 10 years old.

**Fig. 4:**
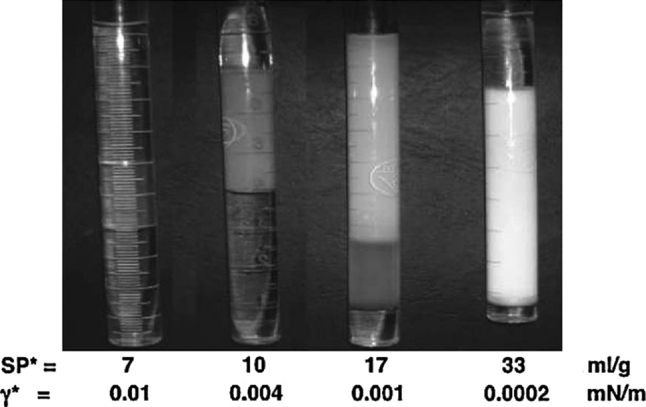
Aspect of three-phase behavior optimum systems with different performance levels indicated as the solubility parameter SP* and the interfacial tension γ* at the optimum formulation

Figure 4 shows several cases of WIII systems close to optimum formulation with the same concentration of different surfactant species, exhibiting different cases from regular to very high performance. The middle phase microemulsion volume (for the same surfactant amount) allows us to calculate the solubilization parameter SP = volume of oil or water at optimum/volume or mass of surfactant, at optimum SP_O_ = SP_W_ = SP_max_, which will be noted as SP*. As previously mentioned, it can be seen that the higher the solubilization the less transparent the microemulsion. The data below the Fig. 4 photograph corroborates the inverse relationship between γ* and SP*.

This relationship was noted a long time ago [33, 34], and it was fully explained as being perfectly normal and even expected with microemulsion models which were relatively simple. Huh’s calculations [35, 36] led to the following equation:

13 $$\gamma {\text{SP}}^{ 2} = {\text{ constant}},{\text{ particularly at optimum}},{\text{ i}}.{\text{e}}.,\gamma^{*} {\text{SP}}^{* 2} = {\text{ constant}} $$

where the asterisk (*) refers to an optimum value in a formulation scan, i.e., a tension minimum, or a solubilization parameter maximum.

This result has been corroborated with most of the cases studied in the past 20 years, with a constant value of usually 0.30 ± 0.05 mN/m when γ is expressed in mN/m and SP in vol/vol.

It is worth noting that the tension and the solubilization are values corresponding to equilibrated systems. There is generally no serious problem with equilibrium attainment for solubilization measurements, but it is critical for tension measurements. In effect, for short time measurement (as often used for technical reasons) big mistakes or artifacts are likely to occur, particularly if the tension is low, as reported elsewhere [37]. A comparison of SP and γ to verify the matching of the Huh relationship could be a safeguard against out-of-equilibrium interfacial tension measurement.

Huh’s inverse relation is extremely useful in practice, because in a formulation scan it is easy and quick to measure the tension if it is relatively high, i.e. when the solubilization is low and difficult to measure, and vice versa. In a typical formulation scan the time saving experimentation consists in measuring the tension in the WI and WII extremes and the solubilization in the WIII cases (and in WI and WII close to WIII if solubilization is high).

As far as the quality of a system formulation at the optimum is concerned, a performance index Perfind has been proposed [38]. It is the cologarithm of the minimum tension γ* in a scan, which may be calculated from maximum solubilization SP* equivalent data, as in Eq. 14. In this relation *C*_S_* is the “height” (wt%) of the three-phase region close to the OW side in a SOW ternary diagram of the Winsor III triangular type [39, 40], or the minimum concentration to attain the single phase behavior at the tail of the fish, Fig. 7b, sometimes called C_X_ [41], which is another way to express the solubilization so that SP* = (100 − *C*_S_*)/2*C*_S_* (≅ 50/*C*_S_* if the solubilization is high).

14 $${\text{Perfind }} = \, - { \log }\gamma * \, = {\text{ 2 log SP}}* \, + \, 0. 5 2 { } = { 3}. 9 2 { } - {\text{ 2 log }}C_{\text{S}} * \, + 2 {\text{ log }}\left( { 1- C*_{\text{S}} / 100} \right) $$

**Fig. 5:**
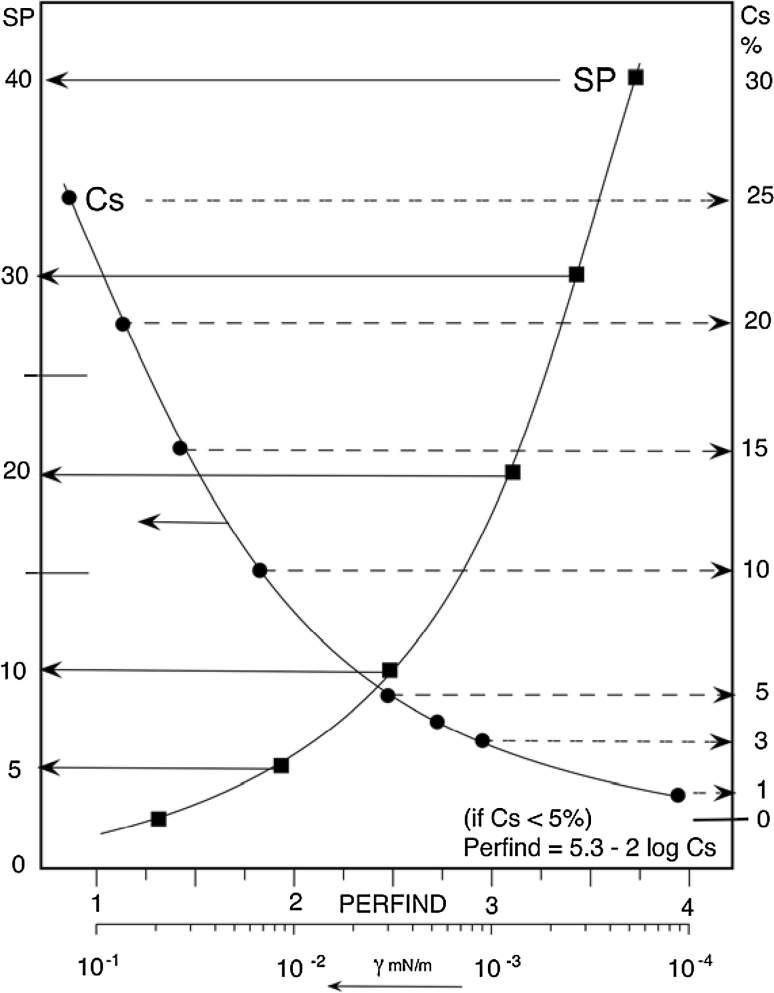
Equivalence of different criteria measuring the performance at optimum formulation according to Eq. [14]

This equivalence of different parameters plotted in Fig. 5 allows us to compare data from different measurements. However, some data have to be carefully reviewed because this *C*_S_* concentration height sometimes includes the alcohol cosurfactant and sometimes it does not.

Many optimum solubilization data have been compared with tension minimum values to build up the trends discussed in what follows. Since the optimum formulation shift with concentration is less variable at high concentration [37, 42, 43], the solubilization data is often more suitable to make a decision, and might lead to a more significant tension expectation, although it is not necessarily the case under enhanced oil recovery process conditions, which tend to be at low concentrations due to the cost and for other reasons.

It may be said that in formulation know-how dealing with ultralow interfacial tension, there are two main ways toward simplification by reducing the number of independent variables.

The first one is to define a quantitative relationship between the variables that result in the optimum formulation condition, so that the actual characteristics of the many components involved could be replaced by a single concept equivalent to Winsor *R*, but calculable in practice. This is what is being dealt with in the present review with the SAD/HLD correlation for pure systems (see Part 1 of this review) and with its equivalent corrected expressions to take into account actual complexities.

The second one is to link the performance of the system as far as the attained minimum tension (or high solubilization) is the primary criterion, to only a few independent concepts or characteristics as the formulation HLD and some performance trends. The guidelines that focus on this goal will be dealt with next, first from the knowledge attained in fundamental studies with pure systems, and then from the know-how accumulated in applied research on real life systems for enhanced oil recovery over the past 35 years.

## Trends to Improve Performance

### Performance Comparison Between Systems

The information gathered in the past 60 years on solubilization and interfacial tension for different purposes has been somewhat disorganized. This is probably due to the fact that it is not easy to clearly understand the trends for a main reason, which is that the independent role of each variable cannot be isolated. Winsor found that, in a formulation scan, the best performance is attained at the optimum formulation of the scan, i.e. it is associated with an optimum formulation where Winsor’s *R* ratio is unity, i.e., *R* = *N*/*D* = 1 or SAD/HLD = 0. Hence, comparisons have to be made between two optimum formulations [44]. Because of the large number of variables and the fact that *R* = 1 or SAD/HLD = 0 is only one relation between all the variables, there are many optimum formulations, because there are many different scans. The question is how to select a better optimum formulation or how to attain the best one of all possible under certain restrictions.

The comparison between two optimum formulations takes place at *R* = 1, but *R* = 1 may be a ratio like 5/5 or 10/10, i.e. with equal but higher or lower interactions on one side and the other of the interface. Half a century ago, Winsor stated that if the interactions were higher on both sides (i.e. *R* = 10/10 in this comparison), then the performance would be better, i.e. a lower tension or a higher solubilization would occur at the optimum formulation [44, 45].

Let’s for instance change both ACN and S with all other variables kept constant. The ACN has nothing to do with the denominator of *R* and the salinity S nothing to do with its numerator. Hence to pass from *R* = *N*/*D* = 1 = 5/5 to *R* = 1 = 10/10, the two variables have to be changed. In this case both ACN and S have to be reduced to increase the interactions on both sides. It is known that by doing so, the performance increases [39, 44, 46]. However it is not known whether it is due to the change in oil ACN or to the change in brine salinity S, or to both together. Similarly in a polyethoxylated surfactant, increasing both the degree of ethoxylation EON and the number of alkyl carbon atoms in the tail of the surfactant (TACN) would increase both interactions as well as the performance according to Winsor’s premise. Again, it may be said that the concomitant change results in a performance improvement, but not what is the specific role of EON or TACN change, and more analysis is required.

An increase in ACN means a reduction in the interaction of the surfactant tail with the oil phase (because of a higher increase in self interaction between oil molecules) as discussed elsewhere [44] and in Part 1 of this review [1]. Consequently, it would be associated with a reduction in Winsor’s *R* numerator (which should be concomitant with a reduction in the denominator) and thus with a decrease in performance. The same usual result is attained when the salinity is increased (with a reduction in the denominator). These effects occur often, and it is reasonable to say that both ACN and salinity S increases tend to decrease the performance. Nevertheless, this is not completely general since sometimes an increase in ACN or in S is associated with an increase in performance, depending on the other variable used for compensation [47–49]. This might be because Winsor’s premise might suffer some exceptions, as will be discussed later.

**Fig. 6:**
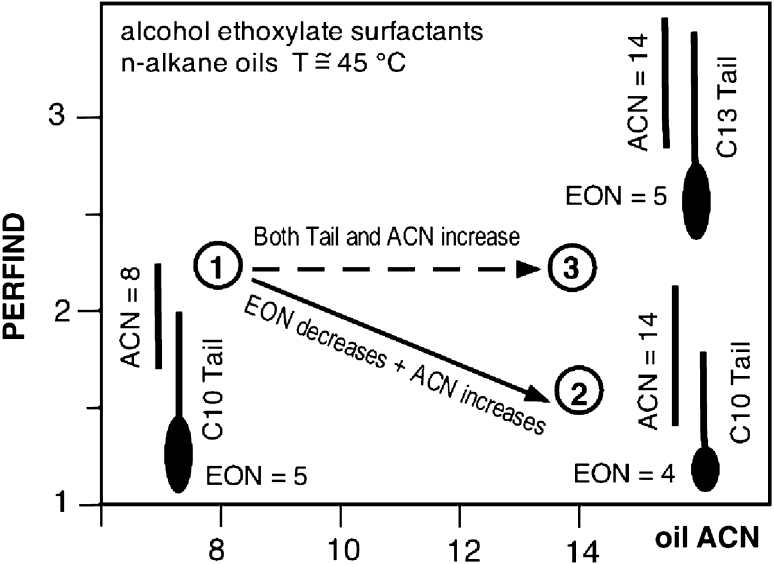
Two typical cases of variable double change to pass from an optimum formulation to another one and compare performance

Hence the concomitant changes might not be a completely foolproof way to get an absolute trend. Nevertheless, this double change method is by far the most convenient and useful method, and it is considered as a good hint in most cases, and it is recommended to be used as the first technique. The analysis of the effect of concomitant changes consists in starting at Winsor’s *R* = 1 and to end at another case of *R* = 1 after two successive changes in formulation. The first one is to change *R* from 1 to some other value by changing a variable that alters the numerator *N* or denominator *D* of *R*, and then, in the second step to change another variable also able to alters *N* or *D*, but this time in the opposite direction, to return to *R* = 1. In such a double change *N* and *D* are at the end equal or different from their original value, depending on whether the two changes are both on the numerator or denominator, or one in the numerator and one in the denominator. Figure 6 indicates examples of the two types of associated changes [40], using data with pure nonionics [20].

Figure 6 indicates the two changes 1 → 2 are a decrease in EON (less interaction on the water side) and an increase in ACN (less interaction on the oil side) with a resulting Perfind decrease, i.e. a performance decrease. In Fig. 6 the two changes 1 → 3 are an increase in the surfactant tail length, hence an increase in interaction on the oil side, and an increase in ACN, i.e. a decrease in the interaction on the oil side. This second change is equal but opposite to the first change, because the numerator has to return to the original value since the denominator has not been altered. In this case the performance is not changed.

This analysis was carried out for all possible concomitant dual changes [39, 40, 50] and it was found that the performance is changed only when both numerator and denominator are changed one way or the other, although a few more complex cases arise when a variable alters more than a single term, which is the case with the temperature.

This formulation variable double change is essentially like moving along the optimum formulation line in a bidimensional map, e.g. in the projection on the S-ACN minimum tension line illustrated in Part 1 review Fig. 3c graph and Fig. 4 graphs. In a large part of the published data on optimum formulation, the performance is not mentioned in the phase behavior plot. Nevertheless, in some cases the three-phase zone located about the optimum formulation line is indicated, and it is qualitative information on the performance.

**Fig. 7:**
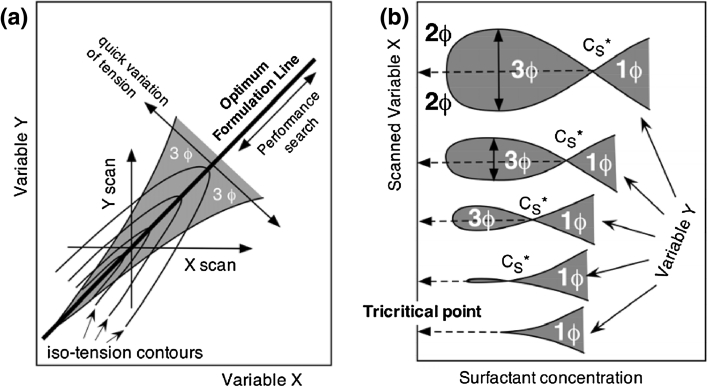
Evolution of the three-phase zone in two bidimensional plots showing variation of the phase behavior according to the change in performance. **a** Two formulation variables plot at constant surfactant concentration; **b** fish diagram versus two formulation variables

Figure 7 indicates the typical aspect of such phase behavior in two classical types of plots. In both cases, the direction along which the width of the three phase zone decreases indicates an increase in performance with the concomitant change of two formulation variables.

If the two scales are taken as being identical according to the SAD/HLD formula as it is the case in Fig. 7a, then the optimum formulation line is the main diagonal, and it is along this line that the search for a better performance should be carried out. The width of the three phase behavior zone about this line is a quantitative measurement of the performance according to Eq. 12 provided that the situation of pure surfactant systems applies, which is the case at least approximately in simple systems.

When the width of the three-phase zone tends to zero, it indicates the approach to a critical point, i.e. a tricritical point at the end of a three-phase cusp [50–52]. In the fish diagram a tricritical point happens when the fish gets completely flat [53], and the three phases zone vanishes, although not necessarily with the solubilization C*_S_ becoming zero (see Fig. 7b). It is expected from theory that the width of the three-phase behavior varies as the HLD distance from tricritical point to the power of 3/2 [53]. It has been shown that the approach of a tricritical point may be carried out by changing a single variable towards the value of this variable at the tricritical point [41] provided that something else is changed to keep the optimum. In other words a concomitant variation of two variables is also required. If the tricritical point is not easily attained with alkanes, because ACN = 5 is the shorter liquid one, it is with polar oils like aromatics [41], or ethers [54]. Moreover it can be approached by changing the head and tail of the surfactant [41] or, as in most practical cases, only the head or the tail of certain species in a mixture of surfactants, with an appropriate way to determine the path toward the improvement. In enhanced oil recovery real cases, the oil EACN, brine salinity and temperature are essentially fixed, and if a phase behavior study is carried on, it is with a surfactant mixture with complex influences, and it is not in general obvious how its composition might improve the performance.

Before discussing the trends that arose from the know-how generated over the past 20 years, i.e., how to improve the performance by changing the surfactants, and eventually by mixing them in intermolecular or intramolecular ways, the basic tendencies will be extracted from some data concerning a very simple ternary system with pure components.

### Simple Relationship Between Performance and Formulation for a Very Simple Surfactant–Oil–Water Ternary System

In Part 1 of the review it was seen that the condition between the formulation variables was a linear relationship with simple surfactant–oil–water systems made of essentially pure substances. The data on interfacial tension available for systems containing pure oligomers of ethoxylated *n*-alcohols, unsalted water and *n*-alkane versus temperature is not distorted from phenomena like fractionation of mixtures, and exhibits a good linearity for a temperature range from 20 to 60 °C. It is thus a good candidate to test a basic relationship between performance and formulation.

With the data provided by Sottman et al. [5], the correlation to attain the optimum formulation for *n*-alcohol ethoxylate pure oligomers characterized by their head group characteristics as the ethylene oxide number (EON, “*i*” in the CiEj formula of the surfactant) and their tail measured as SACN (surfactant *n*-alkyl carbon number, “*j*” in the CiEj formula) with pure water and pure *n*-alkane (ACN) is as follows in the absence of alcohol and electrolyte [55].

15 $${\text{HLD }} = \beta\,-\,{\text{K ACN}} + \, c_{\text{T}} \left( {T - 2 5} \right) \, = \, 0 $$

with *K* = 0.15 and *c*_T_ = 0.05 ± 0.01 for this kind of EON/T range according to the reported studies [55, 56]. The characteristic parameter β may be split into two terms [55]

16 $$\beta = \alpha \,-\,{\text{EON}} $$

where α refers to the effect of the hydrophobic part, and has been found [1, 57] to increase proportionally to the number of carbon atoms of the straight tail, which has been called SACN (surfactant *n*-alkyl carbon number) according to

17 $$\alpha /K \,=\, {\text{Constant}} + 2.4\;{\text{SACN}} $$

where the constant depends on the structure of the surfactant and the reference temperature. The correlation for the optimum formulation for a system containing these oligomers, *n*-alkane and water is found to be as follows for the used data [5] with *c*_T_ = 0.05 (± 0.01) and *C* = 2.0 (± 0.1).

18 $$2.0 \,+\, 0.34\;{\text{SACN}}\,-\,{\text{EON}}\,- \, 0.15\;{\text{ACN}} + \, 0.0 5 { }\left( {T - 2 5} \right)\,=\, 0 $$

**Fig. 8:**
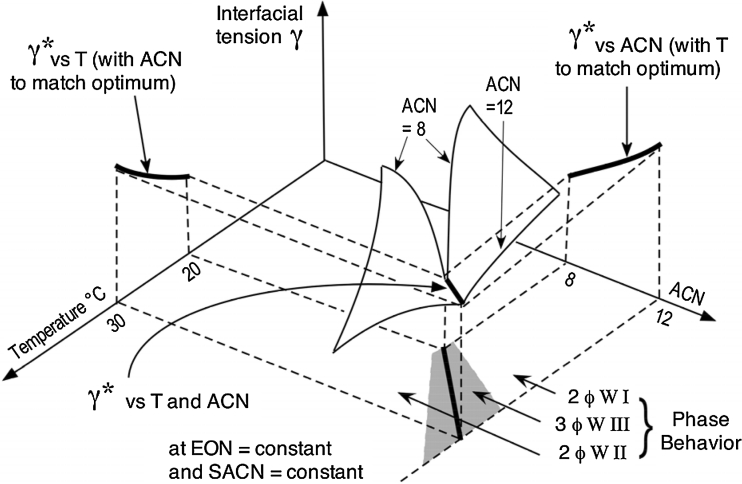
Variation of interfacial tension versus two independent variables (*T*, ACN) for a given ethoxylated alcohol surfactant with the head and tail group defined by EON and SACN

The minimum tension at the optimum which is taken as the performance estimate, written here as γ* to indicate it is an optimum formulation value. In this case, γ* depends on four formulation variables, three of which are independent, with the fourth one value taken to satisfy the HLD = 0 equation (18). The typical tridimensional graph shown in Fig. 8 is essentially similar to Fig. 3c of the first part of this review [1], but with variables used in a nonionic system.

Figure 8 indicates how the tension value may be plotted as a function of two independent variables (here *T* and ACN) when the two others (EON, SACN) representing the surfactant are held constant.

For each scan along the first variable (*T*) there is a minimum tension γ* and when the other variable (ACN) is scanned this minimum point generates a minimum tension line indicated as the bold line in the depth of the valley shaped γ surface. This minimum tension is also found by scanning ACN at constant temperature and the bold line results from scanning *T*. This bold line is the geometric locus of γ* versus the two (*T* and ACN) variables. It is projected as the typical optimum formulation line in the bottom plane, in which the three-phase zone is indicated as shaded. The locus γ* line may be also projected on the two vertical planes to indicate the optimum formulation line versus T (at ACN matching optimum) and versus ACN (at *T* matching optimum).

**Fig. 9:**
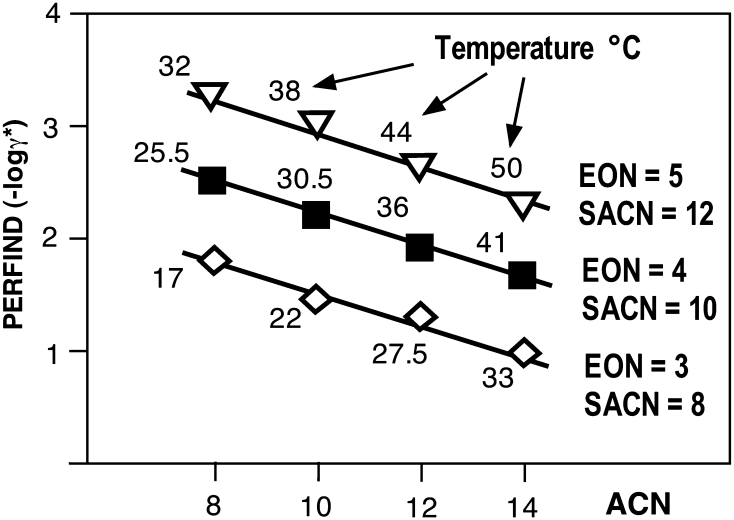
Variation of performance index versus ACN, at *T* adjusted to fit optimum formulation for three *n*-alcohol ethoxylate pure surfactant oligomers defined by their head (EON) and linear *n*-alkyl tail (SACN)

Figure 9 indicates such projected lines, which indicate the performance index (Perfind = −log γ*) plotted versus ACN with the temperature value matching the attainment of an optimum formulation according to Eq. 18. The three lines shown correspond to various surfactant characterized by their head (EON) and *n*-alkyl tail (SACN) included in the used data [5].

The variation of the performance at optimum versus ACN for the three surfactants perfectly matches a straight line. A similar straight line variation of Perfind is found versus any of the four variables, provided that two other variables are constants and the last one is selected to match HLD = 0. It means that Perfind varies according to a very simple relationship that could be very useful to predict the effect of the variables. For this simple case, the projection of the logγ* line in any of the three base planes of Fig. 8 (γ-T, γ-ACN and ACN-T) is a straight line, the bottom plane one corresponding to the classical HLD = 0 equation (18) in a T-ACN plot [55].

However, the way to handle this result is not straightforward because, as seen previously, the comparison between two cases at the optimum involves at least a change in two variables. As a consequence the proper plot to make comparison should involve two variables.

**Fig. 10:**
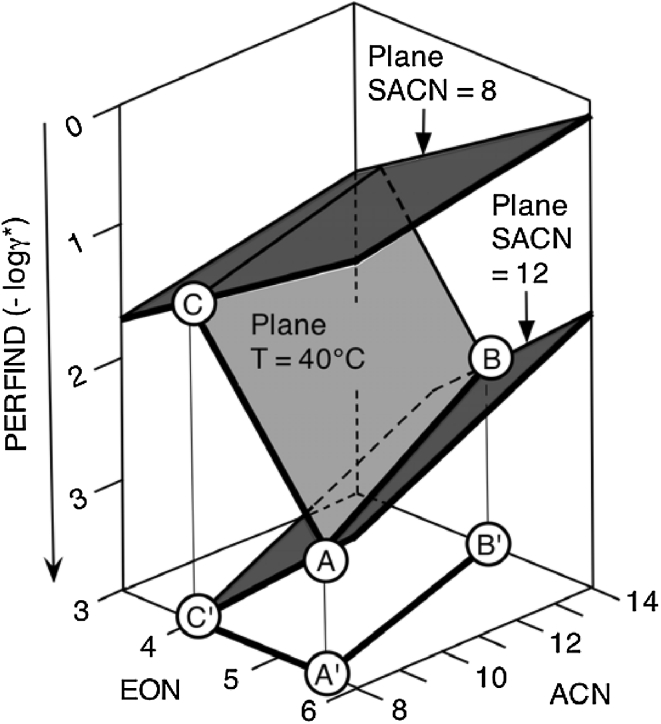
Performance index plot versus EON and ACN. The (*rather* *horizontal*) planes correspond to a constant value of the surfactant alkyl tail (SACN = 8 and 12) at the temperature required to match an optimum formulation. The (*rather vertical*) plane is at constant temperature (*T* = 40 °C) with a tail length (SACN) value to attain optimum formulation

Figure 10 exhibits the results of published data [5] in a tridimensional plot, in which the performance index (−log γ*) is plotted versus EON and ACN.

All the data points corresponding to a given surfactant tail (SACN) are found to generate a plane which is the locus of the Perfind (as −logγ*) vs EON/ACN at SACN constant, with *T* matching the value to attain HLD = 0. Two of these planes are indicated in Fig. 10 for SACN = 8 and 12. A third plane in between (not shown for the sake of simplicity) is found for SACN = 10.

The longer *n*-alkyl tail (SACN = 12) corresponds to the lowest plane, and thus results in a better performance according to the downward scale for increasing Perfind. However, it is worth noting that the planes are neither horizontal nor parallel and consequently, other variables are likely to play some role.

Figure 10 also indicates the locus of Perfind versus EON/ACN at *T* constant (here 40 °C), with SACN matching the required value to attain optimum formulation, which is also found to be essentially a plane, in this case a slanted and almost vertical plane. Other planes (not shown) found at other temperatures, are almost parallel to the *T* = 40 °C one. Since the coefficient *c*_T_ is not absolutely constant versus EON and *T* [56], the surface is not exactly a plane but is very slightly bent, with no significant importance for the following discussion in which it is supposed to be a perfect plane.

The intersection of two planes, one at SACN constant (SACN = 12) and the other at *T* constant (*T* = 40 °C) results in a straight line (bold line) AB that shows the variation of the Perfind vs EON/ACN at both SACN and T constant. Along this line the variation of ACN from 8 to 14 is matched with a variation of EON from 5.6 to 4.7 as indicated between points A and B. This double change fits the HLD = 0 equation, and when the segment AB is projected to the bottom plane it becomes segment A’B’, whose equation is:

19 $$\Updelta {\text{EON}}\,=\,-\, 0.1 5\;\Updelta {\text{ACN}} $$

This double variation (increase in ACN and decrease in EON to keep optimum formulation at constant SACN = 12 and *T* = 40 °C) results in a decrease in Perfind from 3.1 to 2.6.

This data plot also allows to analyze the double effect of EON and SACN, i.e. of the tail and head of the surfactant at constant ACN = 8 (a vertical plane in Fig. 10) and *T* = 40 °C (the slanted almost vertical plane). Figure 10 shows that the intersection of these two planes is a straight line from point C in the SACN = 8 plane at EON = 4, to point A in the SACN = 12 plane at EON = 5.6. This dual variation results in a change in performance from 1.4 to 3.1, i.e., a considerable increase, with both an increase in the head (EON) and tail (SACN) groups of the surfactant.

**Fig. 11:**
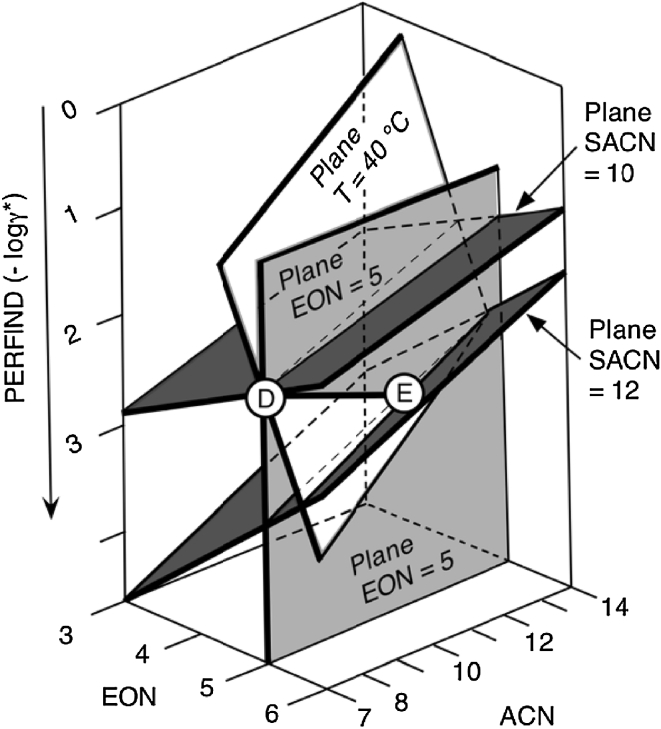
Performance index plot versus EON and ACN. The *planes* crossing along line DE correspond to a constant value of the surfactant head (vertical at EON = 5) at temperature required to match an optimum formulation, or to a constant temperature (*T* = 40 °C) at an EON matching the optimum

The dual effect of ACN and SACN can be seen in Fig. 11 by intersecting the slanted plane (at *T* = 40 °C) and the EON = 5 vertical plane. This produce a straight line from the point D (ACN = 7 and SACN = 10) to the point E (ACN = 11.2 and SACN = 12) with a Perfind variation from 2.2 to 2.8. Consequently, it may be said that in this dual change the double increase in both ACN and SACN increases the performance. It is worth noting that the Perfind increasing trend is the same as in other cases for the effect of an increase in SACN. However, it is the opposite trend for the increase in ACN, which in most cases is unfavorable for the performance, as in the AB change in Fig. 10.

This discrepancy between the two dual changes clearly indicates that it cannot be said that the performance varies one way when only one of the formulation variables changes, even in this simple ternary system case. Any trial to alter performance should involve at least two variables, and of course some dual changes are more effective than others, and in practical complex cases concomitant changes in 3 or 4 variables at the same time might be even better, as will be shown in the next sections.

The fact that the Perfind varies linearly in a 2D optimum formulation plane as shown in previous figures is important and provides guidelines for the practitioner seeking to improve formulation.

**Fig. 12:**
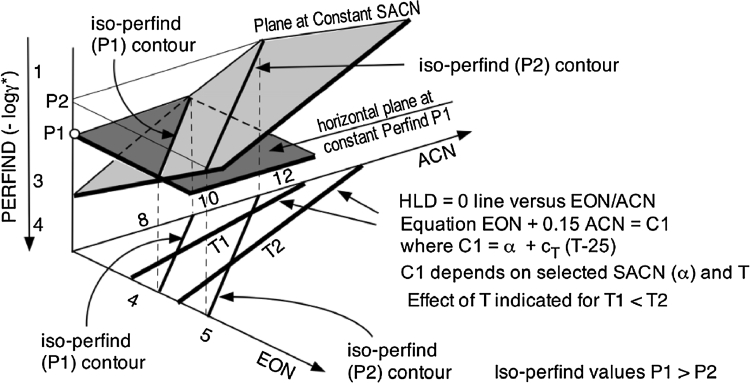
Optimum formulation correlation lines (at constant SACN and T) and iso-Perfind contours (at constant SACN and constant Perfind) in the EON/ACN bidimensional plane

Figure 12 shows the intersection of a horizontal plane at constant Perfind (iso-Perfind cut) with a constant SACN plane in the 3D space with formulation variables EON and ACN, as in previous figures. The intersection of two planes is of course a straight line, which is called an iso-Perfind contour in what follows. In Fig. 12 two contours at Perfind values *P*1 and *P*2 (*P*1 > *P*2) are indicated in the shown constant SACN plane, as well as projected in the bottom EON/ACN plane. In this bottom plane the HLD = 0 correlation is indicated as a straight (bold) line at constant SACN for two temperature values *T*1 and *T*2 (*T*1 < *T*2) according to the typical trends reported a long time ago [47, 55, 58]. It is worth noting that in the EON/ACN bottom plane the iso-Perfind contours are at constant SACN and constant Perfind, with the temperature matching the HLD = 0 condition, while the optimum formulation lines are at (the same) constant SACN and constant temperature.

The values of ACN and EON indicated in the axes do not match the exact experimental data but are illustrative and consistent with the trends.

The performance variations along an optimum correlation line at constant *T* is such that Perfind increases with a dual change including a decrease in ACN and an increase in EON. The displacement of the optimum formulation line when the temperature increases (for instance from *T*1 to *T*2) results in performance diminution along increasing EON at ACN constant, and by increasing ACN at EON constant.

This also shows that it is not because EON increases that there is always an associated increase in performance, as it is often the case. At constant ACN, the performance diminution due to the increase in temperature is more important (for the performance variation) than the usual improvement due to an increase in EON. Actually, this may be explained by the fact that the actual hydrophilicity of the EON head group tends to decrease as the temperature rises because of the dehydration of the polyether head group. Consequently, more (less efficient) ethylene oxide units are required to balance the interaction on the oil side.

Such counterintuitive results have also been found with ACN and salinity variation with anionic surfactants [59], and even if they are not logical according to Winsor’s premise discussed earlier, they have to be considered as tricky but advantageous exceptions to improve performance.

The main conclusion concerning Fig. 12 results is that all iso-Perfind contours are straight lines, which means that there is no maximum nor minimum in Perfind anywhere in the space.

The change in formulation to improve performance is a dual change of two variables in an optimum formulation plane. Of course, the best path of change in this plane that maximizes the improvement is to move along perpendicularly to the iso-Perfind contours. Because of the shape of the iso-Perfind contours, the improvement path will proceed not towards a maximum, but indefinitely in a direction until a limit is found, such as the insolubility of the surfactant in oil or water. This means that in such a case, the improvement of performance will be, of course, linked not only with the proper path (perpendicular to the Perfind iso-contours) in the formulation space, but also with the shift of the limits of formation of a surfactant–oil–water system which present no problem.

As far as the temperature influence is concerned, this is for instance, the solubility in liquid indicated by the Kraft point for ionic surfactants or the cloud point for nonionics. In such a situation where there is a limit, the solution will be to modify the system in order to displace the boundary further. This is what actually has been done to avoid precipitation in realistic systems with many variables, as discussed in the next sections on systems for enhanced oil recovery.

It will be seen in the third part of this review that complex systems with surfactant mixtures, are able to produce synergies, which likely result from non-linear effects that induce minima or maxima of performance. Consequently, it may be said that the results reported here for the behavior of *very simple* ternary systems discussed in the present section, are only guidelines. However, they are important because they indicate what are the main trends to take into account in order to eliminate difficulties in the most complex cases.

### Winsor’s Intuitive Premise on Surfactant Structure Effect: Success and Limitations

In what follows, the tested realistic systems are more complex, in particular with many species of all components since very pure products cannot be used in practice. These real systems exhibit more or less deviation from the linear optimum formulation relationship and non-constant parameter values with changing surfactant concentration or water-to-oil ratio. However the eventual lack of accuracy was not a problem in the first studies in enhanced oil recovery, which were qualitative rather than quantitative, when a progression of the understanding of the phenomena took place in the past half-century.

The general trend to improve the solubilization and tension performance according to Paul Winsor’s premise, suggested more than 50 years ago, is to stay with the optimum formulation and to increase the interactions of the surfactant with both oil and water. In enhanced oil recovery applications, the crude EACN and the brine salinity, as well as the temperature, are fixed in most cases. Hence, the changes in interactions to be carried out have essentially to do with the surfactant(s) and cosurfactant, which have to be selected to take into account the restrictions, but taking into account the EACN, S and T values, which are determinant for the final result.

The simplest way to increase the surfactant interaction with oil is to increase the tail length, particularly if it is a linear alkyl radical with the hydrophilic group at an end. If the correlation for optimum formulation with pure oligomers of ethoxylated *n*-alcohols (CiEj) is taken at the reference temperature, the surfactant characteristic parameter β may be written according to Eq. (18) as β = 0.34 SACN − EON, where SACN is the surfactant *n*-alkyl carbon number, i.e. the length of the *n*-alkyl tail, and EON the number of ethylene oxide groups in the head. A head/tail dual change that would not change the formulation implies that the surfactant parameter β keeps the same value, i.e. that the changes in EON and SACN are equivalent according to the β expression. It means that the increase in the tail by three carbons atoms is just the opposite of an increase in the head by adding one ethylene oxide group. Consequently, C6E2 should have the same characteristic parameter β than C12E4, a fact confirmed in the literature by the exhibition of the same optimum temperature with a same alkane [20, 60]. The two species thus have the same characteristic parameter in formulation issues. However, the effect of the same increase of the interactions in both sides of the surfactant is extremely significant on the performance index Perfind that increases from 1.15 to 3.7.

Some exact concomitant increase in interactions on both sides is quite a coincidence with integer EON and SACN values, and as far as we know there is only one other case in which two very pure surfactants have been found to exhibit exactly the same optimum formulation. It is the case of the sodium salt of dodecyl sulfate and of the *n*-acetyl α-amino eicosanoic acid, the second one with a longer tail and a double head and with a Perfind of 2.3 compared to 1.2 for the SDS [40].

In the data published for pure products, the two effects of integer variations of carbon atoms in the tail and ethylene oxide in the head are not in general exactly compensated and a third formulation variable has to adjust the HLD = 0 formulation, though in a contribution which does not significantly change the trend. For instance, in the CiEj data [5] the series of systems at different temperatures to exactly adjust at the optimum formulation (at the minimum tension with *n*-octane) C4E1 (17 °C)/C6E2 (7 °C)/C8E3 (16 °C)/C10E4 (25 °C)/C12E5 (33 °C) produces a Perfind improvement sequence 0.7/1.1/2.0/2.7/3.4 respectively, i.e. the head–tail almost equal change dominates the effect over the slight temperature change.

The same trend is clearly evidenced if some intermediate data are interpolated between pure species, for instance for C_i_E_j_ and octane at 25 °C the sequence of virtual species with intermediate EON such as C8E3.3/C10E4.0/C12E4.5 would exhibit a corresponding Perfind increase 1.70/2.55/3.4.

Most of the data available in the literature concern commercial mixtures, where the tail and head are described as some average, which is not often known accurately and whose distribution may be different from one case to another, and consequently would bring other effects as will be seen later. In most reported cases the changes are not carried out with equivalent changes of the surfactant on the oil and water sides, but with compensations easy to handle in practice. For anionic surfactants, the change is usually of the linear tail length through a molecular weight variation in alkyl–aryl sulfonates compensated by a change in salinity on the water side or in ACN on the oil side. Although the variation of ACN is limited by the liquid state of the alkanes, it can be extended on the low value side with polar oils whose EACN may go down to negative values [1]. This is not inconvenient to evaluate the surfactant characteristic parameter change, but it may be misleading to evaluate the performance change [39]. For polyethoxylated surfactants what is used is a change in average EON compensated by a change in surfactant tail length or oil ACN. The last is appropriate to evaluate the performance change, but it also produces a variation in partitioning which may have a large influence on the value of the performance.

Let us see what kind of well-supported data are available in the literature. The most significant report on the influence of the surfactant tail was with nonylphenol ethoxylates, compensated with EON or ACN [30], but unfortunately plotted against ACN instead of EON which would be more directly significant as far as performance is concerned. The comparison versus tail length (Tail Alkyl Carbon Number TACN) when the tail is not necessarily an *n*-alkane and EON at constant ACN, indicates for these commercial surfactants the same relationship as for the pure CiEj, i.e. β = 0.38 TACN − EON, with a very slightly different coefficient eventually related to the fact that the tail is not necessarily a *n*-alkyl radical.

As far as the performance is concerned, the following series of systems with alkylphenol ethoxylates, hexane, alcohol and salt: C8ϕE6.0/C9ϕE6.4/C12ϕE7.5/DiC9ϕE9.7 corresponds to an associated Perfind increase of 1.55/1.7/2.0/2.5 respectively. This is a significant performance improvement, though not as considerable as in the case of a pure linear oligomer CiEj series, probably because of the effect of a branched iso-alkyl tail in the CiϕEj species and the presence of alcohol that tends to reduce the surfactant adsorption per unit area at interface. This indicates that the linear relationship between Perfind and formulation variables reported in the previous section to be found for very simple systems, is probably not valid for surfactant tails different from *n*-alkyl chains.

The data with different alkanes indicates that at constant ACN, the double increase in average head and tail that maintains optimum formulation results in an increased performance. However, when the fixed ACN is raised, the performance data are systematically lowered, about 0.6 units Perfind over the whole liquid range of 10 ACN units. The performance indication (as SP* and as Perfind in the range) is found to be inversely correlated fairly generally with the three-phase behavior width (actually as ΔHLB) for different formulas as far as alkane, alcohol, salt and temperature are concerned. It may be said that commercial polyethoxylated alkylphenols and pure alcohols behave quite similarly. The difference in performance value may be due to the presence of additives such as alcohol cosurfactants, and to the fractionation of extreme oligomer species [43].

The chain length effect of sucrose esters of carboxylic acids has been found to reduce the area per adsorbed molecule and thus alters the performance [61].

Reports on the alkyl–aryl sulfonates and petroleum sulfonates dealing with an increase in tail length compensated by a decrease in salinity, indicate an increase in performance, about 1 unit in Perfind for 3 to 5 carbon atoms added in the tail [32, 35, 46, 62–65], roughly similar to the case of commercial nonionics. However, the results are not easy to discriminate because the counterpart is a change in salinity, and also because of the variety of tail structures, particularly with nonlinear alkyl hydrophobes.

As a general trend, an increase in tail size, compensated somehow on the water side, tends to increase performance, and thus longer tail surfactants are desirable. The main problem in increasing the tail length is the limit of molecular solubility of the surfactant. It may be said that linear tail species are likely to precipitate or form a liquid crystal when the linear *n*-alkyl group reaches C_16_–C_18_ carbon atoms.

There are however several ways to prevent the molecular ordering to take place with species having longer linear tails. The first one is of course an increase in temperature that favors a thermal disorder, or the introduction of electrolytes which under some conditions flexibilize the layer formed of ionic surfactants, but in other could help the precipitation or liquid crystal formation.

The second and early way to avoid the organization of the surfactant molecules was to use a mixture with a “bad” surfactant, i.e. a short head and short tail amphiphile like alcohol, sometimes branched ones. This so-called cosurfactant competes to adsorb at the interface in between the surfactant molecules and push them away from one another. This could be interesting to inhibit the formation of solid phases but in practice it increases the surface area per surfactant molecule and consequently reduces the performance. It is thus avoided if something else can be done to move the limit in a direction that steadily increases the performance.

The third way came directly from the early success to attain ultralow tension with petroleum sulfonates that contain scores of different hydrophobic complex structures. Most alkyl–aryl sulfonates, including the detergency species, are also made from petroleum cuts, and they are likely to contain different tail structures, not only with different lengths but also with various branching and ramification types. This issue was studied thoroughly because the tail branching was found to be very significant both for the hydrophobic contribution of the surfactant tail in its HLD parameter and its resistance to precipitation as seen in Part 1.

Linear alkyl benzene sulfonates with the benzene group in different positions along the alkyl chain were found to exhibit unusual behavior [66]. When the benzene ring shifts from the extremity to the center of the linear chain, the surfactant parameter σ/*K* increases by about 2 units every time the ring moves one carbon towards the center of the chain. This is to compare with the σ/*K* increase in 2.4 units when a carbon atom is added to the tail according to Eq. 17. The data also indicate an unexpected tendency, i.e. that the CMC increases steadily with the tail branching, which means the species becomes more water soluble with a maximum for the isomer with the ring on the fourth carbon of the alkyl chain, i.e. with a tail division in unequal parts.

Other studies reported the same trends but with a σ/K increase of only 1.5 unit per one carbon shift towards the center [67, 68] a slight discrepancy that reveals a problem in calculating the surfactant parameter by extrapolation with a high contribution of alcohol cosurfactant effect in the HLD equation.

A previous report [68] also showed that the addition of a carbon atom on the short or long chain of the branched tail produces quite different effects. This is probably due to a different orientation at the water/oil interface and thus different environment of the two chains, as it was found in micellar packing [69]. The report on the shift in optimum formulation due to the branching in double tail alkyl benzene sulfonates [68] also showed that the minimum tension γ* at the optimum, and thus the maximum Perfind, is not necessarily occurring with the equally branched one which is the more hydrophobic, but for a dissymmetrical branched tail, e.g. the 5ϕC16S species with the benzene ring on the fifth carbon atom of the linear C16 alkyl tail. The occurrence of the lowest tension minimum is probably due to two opposite effects of the tail branching. The first one is an increase in interaction with oil because of the opened double tail that favors contacts with more oil molecules. On the other hand the branching results in an increase in the occupied area per surfactant molecule at the interface because of steric repulsion with neighboring molecules.

**Fig. 13:**
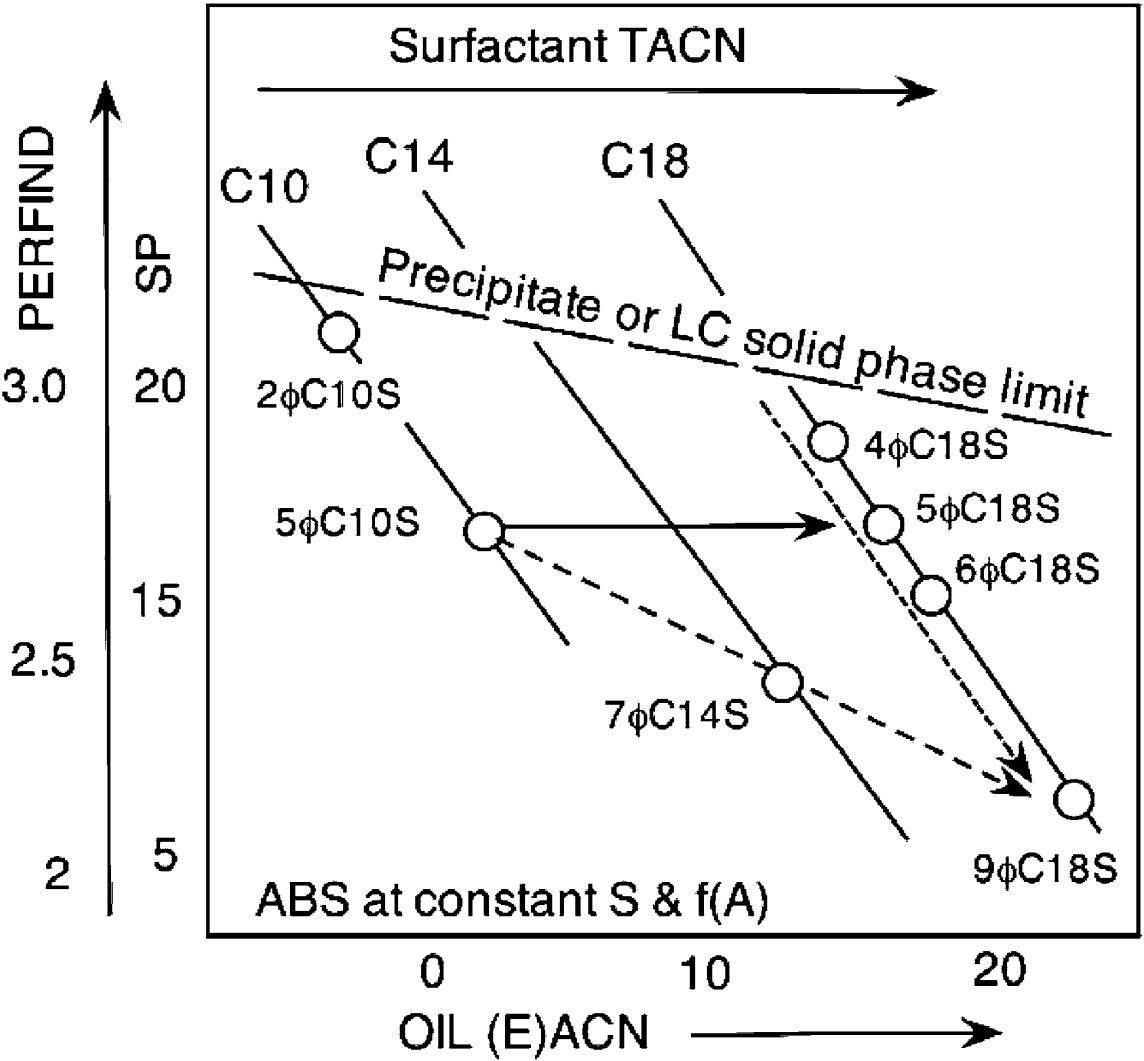
Variation of performance versus oil ACN with the number of carbon atoms in the alkyl benzene sulfonate surfactant linear tail TACN and with the branching, i.e. the position of the benzene ring on the linear tail. Schematics from literature data [31]

This effect of the branching significantly altering the area per molecule seems to be general as indicated in the schematic variations from 4ϕC18S to 9ϕC18S in Fig. 13 showing schematically some trends from another study [31]. It can be seen in Fig. 13 that the perfectly symmetrical double tail (with the benzene ring exactly in the middle of the linear alkyl chain) is not the best species as far as performance is concerned.

The same occurs with isomer-free secondary alkane sulfonates [70]. It may be said that symmetrical tail surfactants require less alcohol to avoid the formation of liquid crystals but they correspond to a higher surfactant parameter, as if they were more hydrophobic, hence they have a lower optimum salinity, in spite of tolerating more salt.

Along the C18 tail line in Fig. 13 plot, it is seen that increasing the branching along the sequence 4ϕC18S/5ϕC18S/6ϕC18S/9ϕC18S reduces the performance. Since the compensatorily preferred ACN for optimum formulation increases, it means that more branching introduces more hydrophobicity for the surfactant tail according to the HLD equation, a clear answer to some questions presented in the literature [71]. Furthermore, an increasing double tail with increasing ACN as seen in Fig. 13 from 5ϕC10S to 9ϕC18S gives less performance. However, it does not necessarily mean that the increasing tail is the reason for this loss of performance, since it may also be attributed to the increase in compensating ACN.

More discussion on the branching and how to use it, is worthwhile, because the branching is probably one of the best ways of forming microemulsions with anionic surfactants in the absence of alcohol, as indicated by the slanting precipitation limit indicated by a dashed line in Fig. 13. This effect is quite general and it has been studied for different surfactants, not only alkyl-aryl sulfonates. Symmetrical alkane sulfonates with double tail 8C16S, 9C18S, 10C20S and 11C22S have their surfactant parameter σ/K that increases 8 units for adding 6 carbons in the double tail (3 on each single tail) [72]. This is a 1.2 unit increase per added carbon atom, much less than the usual 2.4 for a linear alkyl in HLD correlation; may be what counts is the length addition on both sides instead of on each side.

Solubilization data [72] indicates that these symmetrical alkane sulfonates attain an excellent performance at high temperature, with divalent hardness, but that they are likely to produce liquid crystals unless they are used with alcohol, or in mixture with less sensitive species, as will be seen in part 3 of this review.

Multi-tail species as found in petroleum sulfonates or by the synthesis of di/trialkyl benzene sulfonates [73] and di-trichain cationics [74], exhibit good results with some trends similar to branched single tail. Alkyl-aryl sulfonates with several tails, or different basic structures with the sulfonate group(s), e.g. out of benzene ring, have been proposed for enhanced oil recovery [75]. Complex structures such as modified lignin derivatives [76], gemini alkyl benzene sulfonates [77] or cationics [78, 79], exhibit low tension too, probably because of increased packing at the interface.

Of course commercial alkyl-aryl sulfonate species are generally mixtures of species with variable branching [31] with a behavior close to pure products. However the fact that they are mixtures produces a differential partitioning resulting in several effects to be discussed in the next part.

Surfactants without a benzene ring are less hydrophobic and thus more salt tolerant. The benzene ring has been found to be equivalent to 14 linear carbon atoms in the hydrophobic characteristic of the tail [27], i.e. with a sulfonate group placed somewhere along a linear alkyl chain, as in products called secondary alkane or alkene sulfonates [72], alpha-olefin sulfonates [27, 71, 80, 81], or internal olefin sulfonates [64, 65, 67, 82–84].

The performance of these products that always exhibit branching is quite good, but they are generally complex mixtures of very different substances like hydroxyalkane sulfonate, disulfonate, sultone, etc. … and it is not easy to extract a clear information. They will be discussed in the third part on mixtures.

Another way of producing branching close to the double tail case, is the so-called Guerbet chemistry developed more than a century ago. It consists of reacting an alcohol with a proper catalyst to produce a β-branched primary alcohol with twice the molecular weight of the reactant alcohol [85, 86]. This has produced some tested surfactants similar to the double tail species, quite easy to prepare with nonionic or ionic head groups, which were found not to require alcohol cosurfactants [87]. Some studies have compared Guerbet tail sulfates with linear counterparts as far as basic surface phenomena, and found some differences, particularly in aggregation, Krafft point and dynamic tension [88–90]. If the original alcohol is already branched somehow, then the Guerbet dimerization would produce extremely branched tail structures [91] which have also been proposed with even more complexities.

The problem of precipitation or formation of liquid crystals, as well as salt tolerance may also be solved with groups that are less sensitive to hardness as nonionic (polyethoxylates or glucosides) [49, 92, 93] or ionic species more compatible to electrolytes such as polysulfonates, [94] or hydroxyolefin sulfonates. Another way is to combine two parts and two tendencies in the head group as in amphoteric betaine or sulfobetaine [95], ethoxysulfates and ethoxysulfonates [32, 34, 96–99], ethoxycarboxylates [31, 100], to be discussed later as intramolecular mixtures.

Before passing on to the use of mixtures between surfactants or surfactant and cosurfactants, let us see how branching can be used in practice in some compromise with other effects to result or not in a performance improvement.

**Fig. 14:**
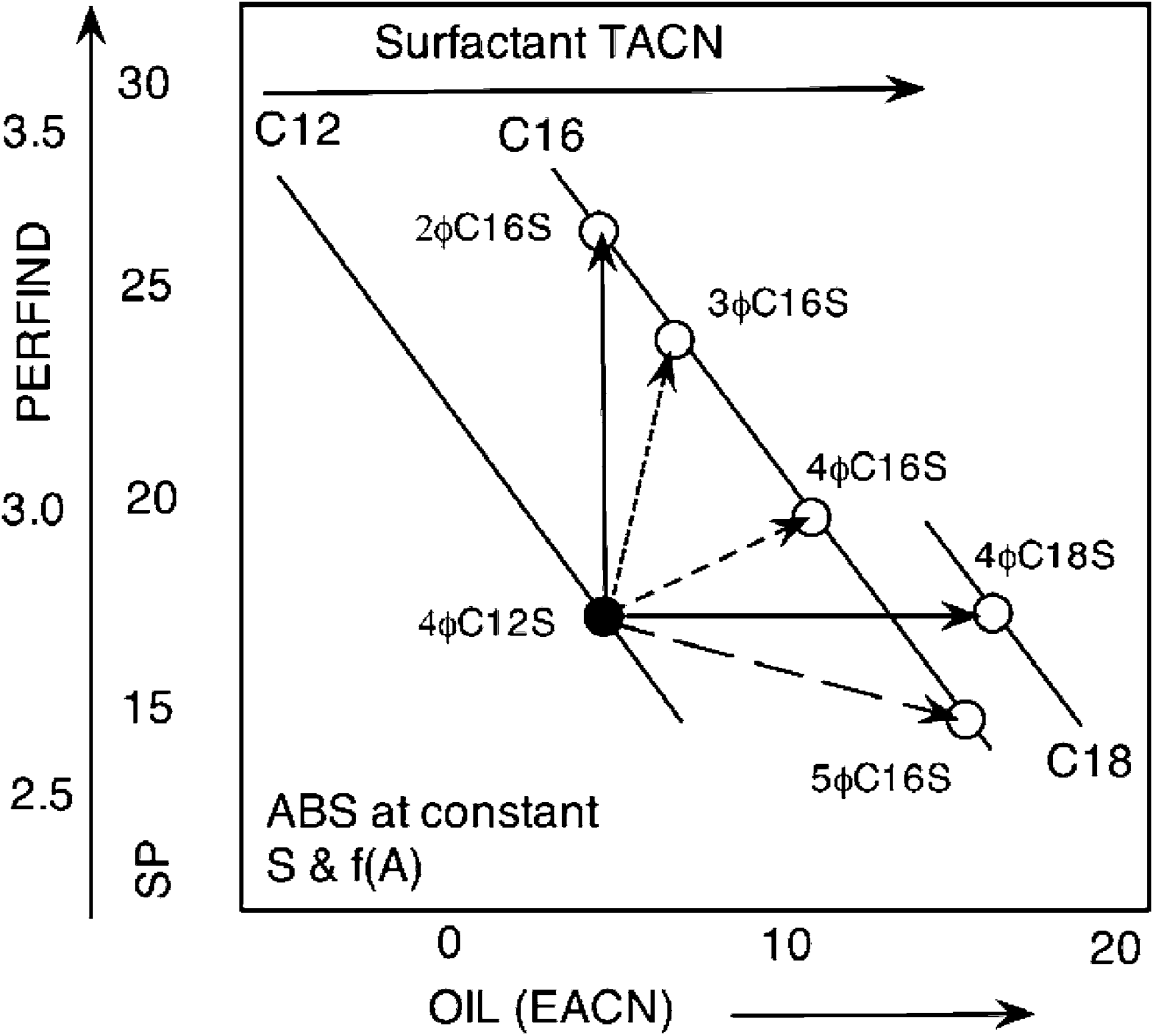
Variation in performance with the number of carbon atoms in the alkyl benzene sulfonate surfactant linear tail TACN and with the branching, i.e. the position of the benzene ring on the linear tail. Schematics from literature data [31]

Figure 14 schematically illustrates a few different cases of change starting from the linear dodecyl benzene sulfonate with the ring in the 4th position (4ϕC12S), i.e. a short chain with 3 carbons and a long one with 8, and a very likely compound or average structure in a detergent product. Figure 14 data extracted from the literature [31] show the change from this starting molecule to other species with different structures as far as tail length and branching are concerned.

From 4ϕC12S to 2ϕC16S, the increase in the tail alkyl carbon number (TACN from 12 to 16) and particularly in the long chain part (8 to 12) increases the interaction between the tail and the oil. On the other hand, the decrease in branching decreases the interaction between the tail and oil to compensate. However, the much smaller short chain (from propyl to methyl) notably reduces the lateral interaction and thus the surface area per molecule and thus increases the performance.

From 4ϕC12S to 4ϕC18S, the large increase in TACN (long chain changes from 8 to 14 carbons) significantly increases the interaction between the surfactant tail and the oil. However, the slight increase in branching slightly decreases it, though not enough to compensate for the previous change, nor to significantly alter the surface area per molecule since the short chain is the same. Nonetheless, an extra compensation is produced by the strong increase in ACN that increases the self-interaction of the oil molecules, so that the tail–oil interaction essentially does not change and the Perfind stays at the same value.

Changes to intermediate species, e.g. 3ϕC16S and 4ϕC16S, produce intermediate situations, and a definite worsening occurs when passing to an even more branched species 5ϕC16S. In a slightly different case [101], the compromise between opposite tendencies resulted in the best Perfind attained with an intermediate dissymetric branching.

This means that the way to handle the optimization is not obvious with pure species, because not only the number of carbon atoms has a significant effect, but also the branching, even in the case of a double tail in which adding a carbon in the short tail could be different from adding it to the long one [68], simply because of a very different environment [69]. Of course this could be even more intricate with mixtures, as will be seen in the next section.

Summing up, it may be said that a large number of publications in the past 30 years have shown that Winsor’s premise to increase performance by increasing the interactions on both sides of the interface is a correct overall tendency, with a limit having to do with precipitation or formation of liquid crystals. A deeper level of studies was thus dedicated to pushing the limit forward, and it was in the direction of three tendencies.

First the extensive use of the tail branching whose characteristics were previously discussed, with some confusion but with a few relatively clear and extensive studies that indicate very complex intricacies which increase the interaction with oil, but not necessarily with a concomitant increase in the performance. The proper combination of changing tail size and tail branching may lead to tricky compromises in the molecular structure depending on the requirements or restrictions concerning a high ACN, a water solubility, a lower tension or a combination of them [31].

Secondly, the use of mixtures as a way to produce molecular disorder and compatibility as well as synergy whose principle will be discussed next. However, all the possibilities are not yet well understood because of highly non-linear rules that require proper experimental guidelines to be discussed in the third part of this review, since there is a wide choice of methods and perspectives.

Third, but first to be studied back in the 1970s, was the use of alcohols or other cosurfactants, with some advantages but also some problems with cost and performance, as will be seen in the next section.

### Intermolecular Mixtures Between Surfactants, Cosurfactants and Other Additives, and Their Inherent Limits

#### Why Mixtures?

Mixtures of a surfactant with other surfactant(s) and with a large variety of additives have been carried out in many practical cases, but very few studies were specific research designed to clear up scientific issues. It is worth noting that in surfactant studies, it is sometimes necessary to use extremely pure surfactants to understand the phenomena, because a very low concentration of an impurity, e.g. less than 0.1 %, can, in some cases, become more important than the main product as far as the effects are concerned. Nevertheless extreme purity is too costly, and irrelevant in practical applications.

Mixtures are used in the real world for two reasons. First of all, most surfactants and all commercial surfactants are mixtures because their fabrication implies the use of raw material blends (e.g. alkyl or olefin groups coming from petroleum cuts) or spontaneously occurring distribution of species resulting from the synthesis (e.g. ethoxylation). The second reason is that formulation with surfactants often requires the adjustment of some property intermediate between those of two existing species, and thus some interpolation is realized through a mixture.

On top of that, an often occurring characteristic of surfactant mixtures is that they result in synergy, i.e. an improvement over the behavior of each of the separated components, which may be due to some increase in entropy or another reason like the formation of a new performing structure. This is, of course, the main reason behind the use of mixtures, and it is worth understanding the different reasons why this happens, which are not obvious.

**Fig. 15:**
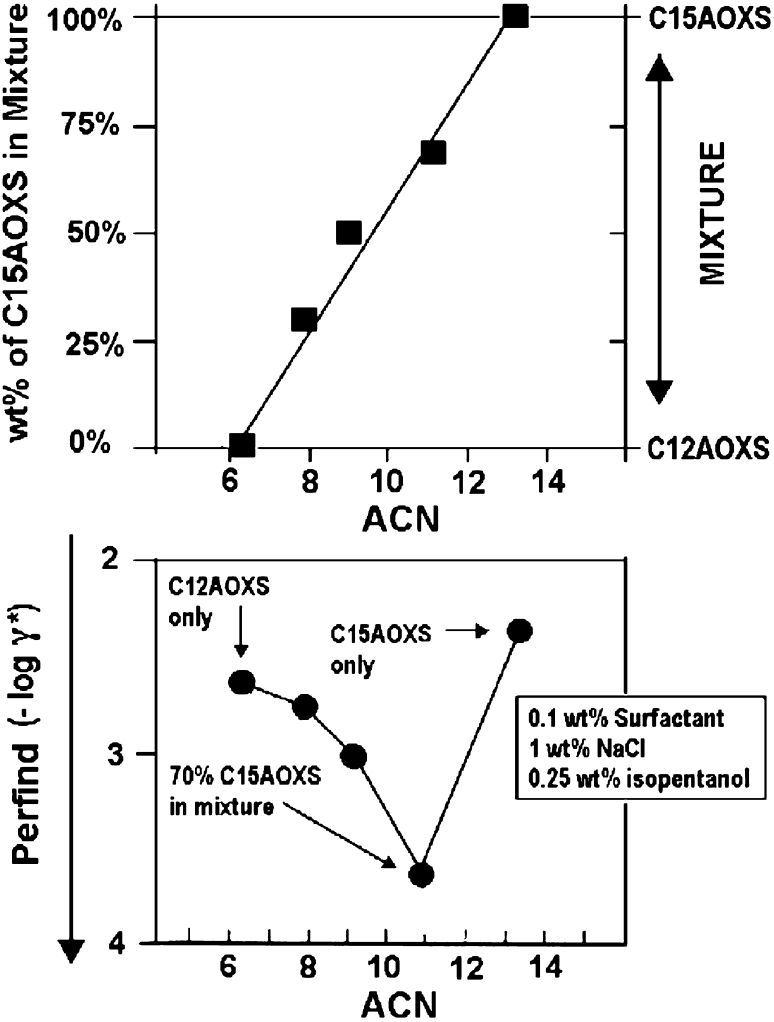
Perfind variation with the composition of a mixture of two close surfactants. Data from [102]

For instance in Fig. 15, which shows the Perfind (−log γ*) for mixtures of two relatively pure alkylorthoxylenesulfonate species (AOXS); it is seen that the Perfind is best with a mixture containing 70 % of C15AOXS and 30 % C12AOXS. It is obviously not because there is more of the surfactant with a longer tail, since the C15AOXS alone exhibits the worst performance of all proportions. It was said [102] that there is a compromise between the increase in the C15 AOXS species in the mixture which tends to improve the performance for a longer tail and the corresponding increase in ACN (changed to keep the optimum along the “ideal” straight line variation of the formulation versus composition in the upper plot) that tends to lower it. The clear improvement in Perfind at 70 % C15AOXS indicates some best compromise in some synergy, since Winsor’s premise would predict no change in performance because the two formulation effects (surfactant tail length and oil ACN) take place on the same side of interface.

In such a case the mixed surfactants are very similar, but there is a significant apparent synergy. It is thus no wonder that even a more noteworthy synergy could be attained with quite different species as it is very usual to find in proposals for enhanced oil recovery formulations from the first years [31, 103–107].

#### Mixing as a Way to Push the Limit of Winsor’s Premise

As was discussed in the previous section, when the surfactant tail and head sizes both increase, the surfactant performance tends to increase, but there is a limit beyond which phase separation can take place because of a solubility restriction. If the surfactant with a bigger size on each side of the interface is replaced by a mixture of two surfactants, one having a large tail and small head (adsorbed onto the oil side of the interface) and another one with a large head and a small tail (adsorbed onto the water side of the interface), the resulting adsorbed pair extend further on both sides of the interface like a bigger single surfactant, and thus will have a higher performance, but without a precipitation problem.

**Fig. 16:**
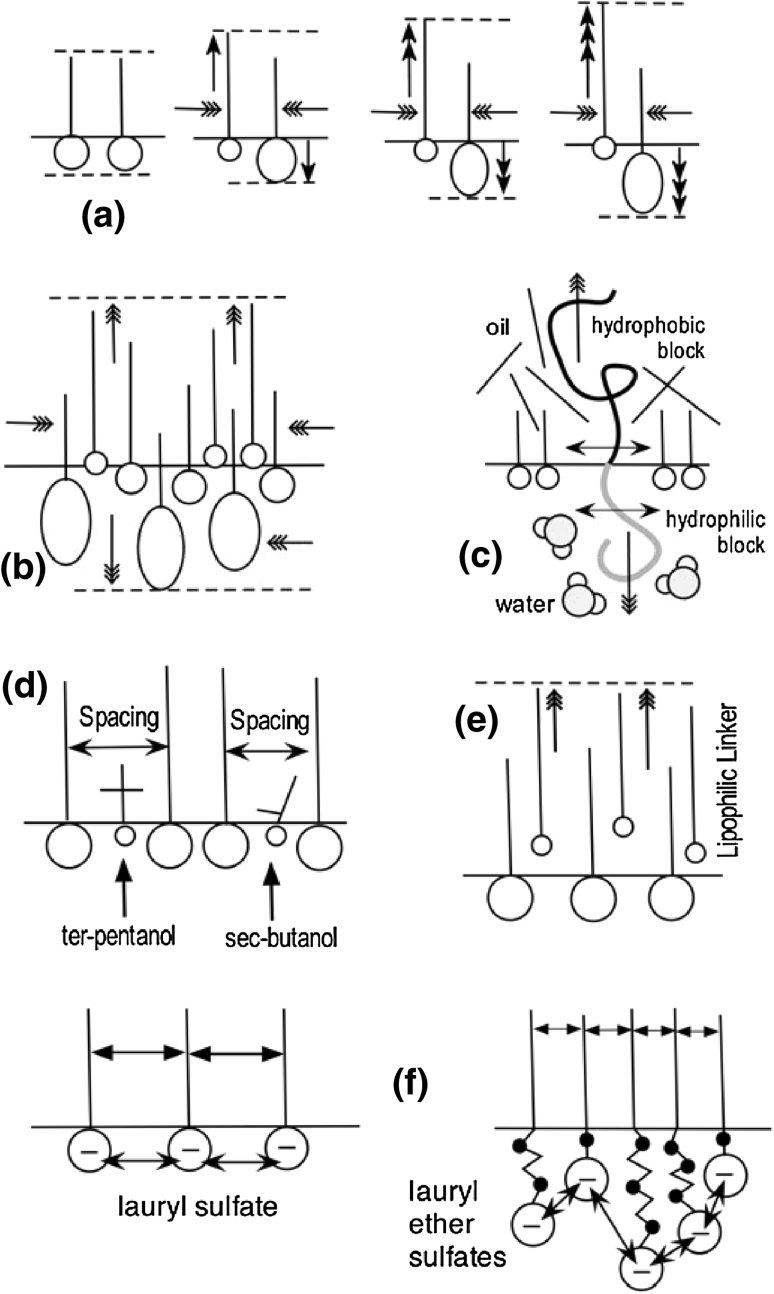
Effect of mixing on the location of the different species at interface: **a** mixture of surfactants with increasing differences from left to right; **b** mixture of more than two surfactants; **c** mixture of a small surfactant and a small amount of a very large one diblock, both balanced; **d** mixture of surfactant and alcohol; **e** mixture of surfactant and lipophilic linker; **f** lauryl sulfate (*left*) and mixture of oligomers of lauryl ether sulfates with an average of 2EO

Figure 16a shows from left to right that this would become more significant as the difference between the two species increases, although as will be seen later, there is a limit due to the selective partitioning of the species in the bulk phases, and thus their lack at the interface.

Figure 16b also indicates this kind of mixtures could result in a lower repulsion between neighboring molecules in both sides and thus a more compact arrangement of the molecules. This would result in a decrease in surface area occupied by the average molecule at interface, a second factor that would increase the performance. This is what has been reported with alkyl phenol ethoxylates [108] by mixing commercial components with different average ethylene oxide number EON to attain the same optimum at interface. The series terC8ϕE5/terC8ϕE3 + terC8ϕE7/terC8ϕE1.5 + terC8ϕE9 with the same TACN, salinity, alcohol and temperature conditions [108] resulted in Perfind variation of 2.0/2.5/2.7. The more different the mixed species, the best the performance improvement, though not necessarily at the same cost, as will be discussed later.

This kind of synergy has been confirmed with different types of mixtures [58, 93, 104, 109–114]. Although this has been known for about 30 years, surprisingly it has been recently mentioned as a novelty that a better synergy may be obtained with a mixture with three surfactants than with two [115]. This is actually obvious if the principle of mixture advantage is well understood [58]. This is probably related with the rule of thumb that, the more complex the mixture, the better it is. However, this is not strictly right, but it indicates a trend which has to do with the spontaneous change to increase the entropy of the system through more disordered arrangement, and this is why the strategy to optimize mixtures, in particular non-linear ones, is extremely important and should be analyzed experimentally with special approaches to find the best, as discussed in part 3 of this review.

#### Other Differences than Formulation

The differences of mixed surfactants may not be in hydrophilicity, but in size and attractiveness or drawback according to Winsor’s premise. This is the case in the mixture association shown in Fig. 16c in which an extra large surfactant so-called amphiphilic block copolymer species [116], e.g. 70 units of isoprene and 100 units of ethylenoxide, which is exactly balanced, is introduced in very small proportions into the system containing mostly ordinary small surfactant species, e.g. C10E4 alcohol ethoxylate, dioctyl sulfosuccinate, or alkylglucopyranoside, which are also balanced. As shown in Fig. 16c the enormous species with a molecular weight 30–50 times the ordinary surfactant, will adsorb at interface as individual molecules completely separated from similar big molecules by the smaller regular ones that occupy an extreme proportion of the area, e.g. 99 %. Because of the extremely large head and tail of the extra big surfactant, its provides a huge increase in interactions on both sides, i.e. a synergy effect that results in an impressive performance improvement, e.g. from Perfind 1.5 to 3, at the very low concentration at which it does not precipitate, e.g. one tenth of the 2 % of surfactant/cosurfactant amount [116–118].

This is just some kind of extreme and partial use of the Winsor’s premise with the extreme dilution trick to avoid precipitation. It is worth noting that the introduction of a homopolymer that corresponds to the half part of this copolymer reduces the performance because it does not influence the rigidity in the right way [119]. Consequently it is the presence of the two amphiphiles, essentially the ordinary one, plus a very small amount of the super-big one that produces the performance boost. However, it is obvious that this kind of mixture can only work if the concentration of the big amphiphilic block copolymer species is low enough to avoid precipitation. It may be conjectured that the association to these two surfactants of a third type with intermediate size (may be at an optimum or may be as a mixture of two whose average is at an optimum) would produce an even better synergy and compatibility to push away the limits of precipitation.

As far as we know, such research study has not been done in a strictly scientific way yet, but the mention of some miracle formulations of this effect (with not so big amphiphilic copolymer) has been reported in propaganda talks (saying it is used with a very low concentration, of course not really for the cost but to avoid precipitation) given in congress clearly indicates than such an association could be extremely performing if it is well handled [115].

#### Synergy Concerning Goals Other than Performance

Some surfactant mixtures might have an interest which deals with a property different from ultralow tension performance, and that could be extremely important for some application like enhanced oil recovery. For instance mixing anionic and nonionic surfactants has several amazing interests that indicate it is an almost compulsory feature.

First of all, this kind of mixture of anionic and nonionic surfactants usually provides tolerance to high hardness and avoids the precipitation of anionic surfactants with divalent electrolytes [29, 47, 120, 121]. Such combination may be rendered too by the incorporation of the two effects in a single molecule [98], i.e. as an intramolecular mixture as will be discussed later.

Secondly, since the influence of temperature on the two types is opposite as seen by a different sign in the SAD/HLD equation in part I of this review, a proper mixture can be made to become insensitive to temperature [120–123], which is particularly important because of the extreme influence of this variable on the phase behavior of polyethoxylated nonionics [124]. Nevertheless, it is worth noting that some nonionic surfactants like glucosides [125, 126] or sucrose esters [127] are much less sensitive to the temperature than polyethoxylates.

The third feature provided by an anionic-nonionic mixture is its potential insensitivity of its interfacial formulation to the dilution or to the change in water–oil ratio [107, 128], which cannot be avoided when the injected slug contacts the reservoir fluids. This effect is due to the fact that the two species have opposite formulation variations [55, 129] with respect to selective partitioning of the different species to the bulk phases [130], which is the main cost problem and the actual limit to the use of mixtures as seen later.

On top of this, it turns out that an anionic-nonionic mixture is more hydrophobic than the separate components because of an association-shielding between the ionic and the polyether groups that reduces the interaction of the combined head group with water molecules [120, 131]. This hydrophilicity diminution at the interface allows to use more water soluble surfactants, which are less likely to precipitate on their own.

#### Alcohol Effects as Cosurfactants

Mixtures have been found to produce structural changes in the association of amphiphilic substances from micelles to liquid crystals and microemulsions [132]. Liquid crystals come from a molecular structuration with a wide variety of arrangements [133]. In enhanced oil recovery the absence of liquid crystals is absolutely required to insure a low viscosity. Since the formulations at which the interfacial tension tends to be low are often close to the formulation in which a lamellar liquid crystal is likely to take place, the elimination of liquid crystals has to be attained through a disorder effect. The first way to do it is to increase temperature, but it is not always possible in practice. The second way is to add alcohols, or other cosurfactants. Other alternatives are to produce other disorder effects due to the surfactant structure (branching) or surfactant mixtures or both.

The alcohols which are not very hydrophilic nor very lipophilic are likely to migrate significantly at the interface and thus to occupy an area with a low interaction with oil and water. This produces disorder at the interface with less interaction with neighboring surfactant molecules and thus no liquid crystal formation. This is very clear in data for alkane sulfonates [29] in which alcohol and temperature effects are compared. As a consequence, the presence of alcohol eliminates the probability of liquid crystal occurrence (and also of precipitation or adsorption) with typical anionic surfactant, particularly relatively pure species [134–136]. Alcohol like *sec*-butanol or *ter*-pentanol have a relatively balanced interaction with oil and water, and thus have essentially no formulation effect. Moreover, their branching insures they pull apart the neighboring adsorbed surfactant molecules.

More hydrophilic ones have a very small effect, while more lipophilic ones like *n*-pentanol or *n*-hexanol contribute to the hydrophilicity-lipophilicity balance at interface and result in a formulation effect included as the term called f(A) or ϕ(A) in the SAD/HLD equations discussed in Part 1 of this review [1] or equivalent way to take into account the alcohol effect [28, 33, 37, 39, 55, 137–142]. This f(A)/ϕ(A) term is easy to measure, but it has not been reported with accuracy in the literature for two reasons. The first one is that the partition coefficient of alcohol between oil and water [143], and of course at the interface, depends somehow of the nature of the phases, and is not independent of the rest of the system. It also depends on the water-to-oil ratio [102]. The second reason is that the alcohol effect at interface also depends on its concentration and sometimes even changes from hydrophilic at low concentration to lipophilic at higher concentration as reported for *sec*-butanol or *ter*-butanol. In all cases the alcohol partition coefficient does not remain constant with the alcohol concentration [143]. Of course this is not very significant in the usual range of use of alcohol (<1 %), but it results in a f(A) term which does not exhibit a straight line variation with concentration above a f(A) value larger than one unit [40] in HLD equation.

When the alcohol is in concentrations high enough for a large part to migrate to the oil phase, it tends to accumulate in the oil close to the interface as all polar oil species tend to do [144, 145] and results in a more hydrophilic oil phase interacting with the surfactant, hence an actual lower EACN of the oil. This of course alters the SAD/HLD equation and complicates things. Additional intricacies have been found because it was shown that the alcohol partitioning between oil and water, changes with the salinity [143].

Since alcohols are competing with surfactants for the occupation of the interfacial area and their interactions with oil and water is much lower, they are likely to penalize the performance according to Winsor’s premise. This is in general the case and the more likely the alcohol is to adsorb at interface, the more it diminishes the performance [39].

However some alcohols, particularly lipophilic ones, were found to reduce the salinity required to attain optimum formulation and thus may be advantageous for some applications. [80]. In other cases a proper amount of alcohol has been reported to improve performance even if it also increases the required ACN or salinity [102, 146, 147]. As discussed previously, the change in formulation from one optimum to another implies the variations of two formulation variables. The alcohol effect can be one and as such it has to be compensated for by another that could be better or worse as far as the performance is concerned. Consequently, even if the alcohol effects on performance are generally unfavorable, there are some exceptions [37, 102].

Mixtures of different alcohols, with an overall f(A) constant effect on optimum formulation, seems to keep the performance constant [39]. This is consistent with Winsor’s premise because the alcohol effect is only variable on the oil side, since the head group is always the same. In such a case, there is neither improvement nor deterioration in the mixing and there is the possibility to adjust an alcohol blending for some other reasons, like adsorption reduction.

A systematic study has shown that alcohols exhibit three different behaviors depending of their tail size [38, 40]. Short very water soluble alcohols (methanol and ethanol) produce a slight hydrophilic contribution, i.e.,  a f(A) term which is equivalent to a slight decrease in salinity. Their adsorption at the interface reduces the performance.

Intermediate alcohols as far as the partitioning between oil and water is concerned (*n*- and iso-propanol, *sec*- and *ter*-butanol, *ter*-pentanol) have practically no effect on formulation, i.e. their f(A) is very small, but they are the ones which adsorb more at interface, thus reduce the surfactant adsorption, and consequently result in the stronger penalty in performance, in particular for branched tail because of lateral steric repulsion [148] as shown in Fig. 16d. The alcohol most used for this purpose is *sec*-butanol, which is assigned a number of carbon atoms in the tail of 3.5 and an almost zero f(A) value.

Lipophilic alcohols which adsorb significantly at the interface, i.e. from *n*-butanol to *n*-octanol, are water soluble only in very low concentration and thus migrate to interface to contribute significantly to the f(A)/ϕ(A) term. However, since they adsorb less than intermediate alcohols [149], their performance penalty is not so severe. In some cases, they might be a good way to produce a benefit, for instance by lowering the salinity.

Very long chain alcohols, say *n*-octanol and longer tail ones, essentially do not adsorb at the interface and consequently their f(A)/ϕ(A) effect on formulation essentially disappears, and most of the amount migrates to the oil phase. It might be deduced that they have no effect on performance, but experience indicates that this is not the case. Very lipophilic alcohols tend to improve the performance, and the effect is stronger when the alcohol is more lipophilic, i.e. when it adsorbs less. This is just contrary to Winsor’s premise because it obviously means that the effect is not due to something that happens at interface. This strong increase in solubilization by adding high molecular weight alcohols like decanol or dodecanol was explained to be due to the segregation of the alcohol molecules (just as a slightly polar oil) close to the interface, but inside the bulk oil phase [145]. The phenomenon, which was called the “Lipophilic Linker” effect, will be discussed next.

#### Lipophilic and Hydrophilic Linkers

The effect was first discovered as the effect of extremely hydrophobic amphiphilic species like nonionic ethoxylates with an average of only one or less ethylene oxide group [108] which cannot be considered as surfactants because they essentially do not adsorb at the interface and thus do not alter the interfacial formulation. They actually migrate into the oil phase in the so-called preferential partitioning phenomenon [130] to be discussed later. Then it was found that long *n*-alcohols from C10 to C18 tail [149] as well as practically any slightly polar oil [150, 151] are likely to accumulate close to the interface in the bulk oil to produce the so-called interfacial segregation effect [145]. Such species generate a very few layers of oil with less hydrophobic characteristics close to the interface, i.e. a lower EACN, and thus the usually higher performance associated to this lower EACN. The lipophilic linker effect depends on the size of both the surfactant tail and the oil molecule, the most efficient lipophilic linker having a size intermediate between the size of interacting parts on the oil side.

This intermediate role was the reason it was called a linker, since it was working just as an adhesive to prolong the tail interaction with the oil, as shown in Fig. 16e. An increase in concentration of the lipophilic linker raises the performance even better if the surfactant alone is better [149]. It was shown that the effective range depends on the surfactant concentration [150], up to a point where no surfactant is available to be extended by a lipophilic linker. Although the lipophilic linker is not adsorbed at the interface, as clearly indicated by its no influence in the optimum formulation, a constant performance may be maintained by a trade off with the surfactant, i.e. an increase of the lipophilic linker concentration does compensate a decrease in surfactant concentration [150]. Studies on interfacial tension have not been carried out, but if Huh’s relationship [35] applies, it means that more lipophilic linker is added, less surfactant is required to get the same performance. This could be useful in practice since the lipophilic linker molecular structure might be more advantageous for a property such as adsorption or precipitation.

**Fig. 17:**
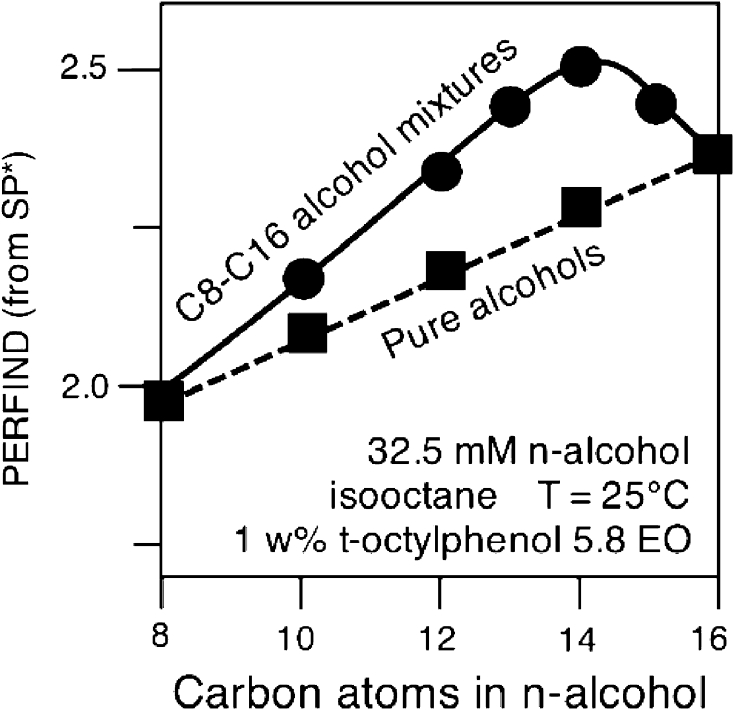
Effect of lipophilic linker pure species and mixtures. Lipophilic linkers are *n*-alcohols at a constant (*low*) molar concentration

Mixtures of *n*-alcohols in C8 and C16, i.e. short and long lipophilic linkers, have exhibited a significant improvement in the performance with respect to pure alcohols as seen in Fig. 17 without any change in optimum formulation (same EON), contrarily to shorter alcohol mixtures [151, 152]. This is probably similar to the synergy found in surfactant species mixtures but this time with lipophilic linker species in the oil bulk close to the interface [149].

The lipophilic linker is an oil, and consequently it also tends to migrate substantially into the oil phase, and even if it accumulates close to the interface, a large part might be dissolved in the bulk oil and thus be lost for the mentioned effect. This preferential partitioning in one of the phases is the inherent limit to the use of mixtures, of linkers or other species. It could be excessive and costly and the effective concentration in the oil is probably too high for enhanced oil recovery applications.

The same kind of effect has been proposed to take place on the water side of the interface, by adding so-called hydrophilic linker molecules which are very hydrophilic amphiphiles, intermediate between surfactants and hydrotropes [153, 154]. The effect is similar, but much less significant in performance improvement because the polyethylene oxide head groups are relatively soluble in oils, and thus do not present critical solubilization problems even at high ethoxylation. Hence the improvement of interactions on the water side (to follow Winsor’s premise) is less important for enhanced oil recovery, unless the additional hydrophilicity they provide happens to be of interest for other issues than low tension, e.g. adsorption. The combination of both lipophilic and hydrophilic linkers might be of interest in some cases [155–157], but probably not in enhanced oil recovery where the partitioning of the species into the bulk phases is a serious loss and costly.

#### Other Additives

Many other additives have been proposed to help in enhanced oil recovery to produce some specific effect as a mixture with surfactants and cosurfactants.

Hydrotropes like short chain mono or di-soaps [158], alkylbenzene sulfonates [159, 160] or alkylpolyglycerides [161] have important and sophisticated effects in complex formulation by influencing the association structures of traditional amphiphiles like liquid crystals precipitates [162]. A mixture of conventional anionic with a cationic hydrotrope has been reported as resulting in considerable synergy, probably because of a significant compaction of pseudo-amphoteric surfactants [163] close to a compact combination of a hydrophilic linker and a surfactant. This has been found to be even better if associated with an extended surfactant [114, 163], i.e. an intramolecular structure discussed next.

Aside from the typical C3-C6 alcohols reported in most microemulsion publications, other alcohols, diols, glycol derivatives, beta naphthol, ethoxylated-propoxylated butanol, alkyl amides and fatty acids have been reported as cosurfactants with systems with a conventional anionic or nonionic polyethoxylated surfactant as well as alkylpolyglucosides [164-176].

Some evidence reported in these studies, clearly indicates a performance improvement, probably equal or similar to a linker effect, although not always. A systematic approach is needed to better understand the effect of some of these non-conventional cosurfactants in order to relate it to a change in the curvature and rigidity in the surfactant layer, particularly in the presence of electrolyte [165, 168].

#### Inherent Limit of Mixture Approach: Selective Partitioning

As was discussed previously, according to the mixture principle of extending interactions on both sides of the interface, the performance related to the interfacially adsorbed material increases as the two species become more and more different. However it should be remembered that adsorbed surfactants are in equilibrium with the surfactant molecules solubilized in the bulk phases. In the case of the mixture, the increase in the hydrophilicity (respectively lipophilicity) of one (respectively the other) surfactant would increase its preferential partitioning into water (respectively oil) and, consequently, more of each surfactant would partition into a bulk phase, and less would adsorb at the interface. The consequence would be that if the performance increases per surfactant molecule at the interface, the performance in the system would decrease at some point. If the performance is measured through the solubilization parameter SP*, two different solubilization values are dealt with, i.e., the interfacial (int) and apparent (app) SP* as follows

20 $$SP*_{\text{int}} = \frac{{\text{amount of oil or water in the middle phase microemulsion}}}{{\text{amount of surfactant in the middle phase microemulsion}}}$$

21 $$SP*_{\rm app} = \frac{{\text{amount of oil or water in the middle phase microemulsion}}}{{\text{amount of surfactant in the system}}}$$

**Fig. 18:**
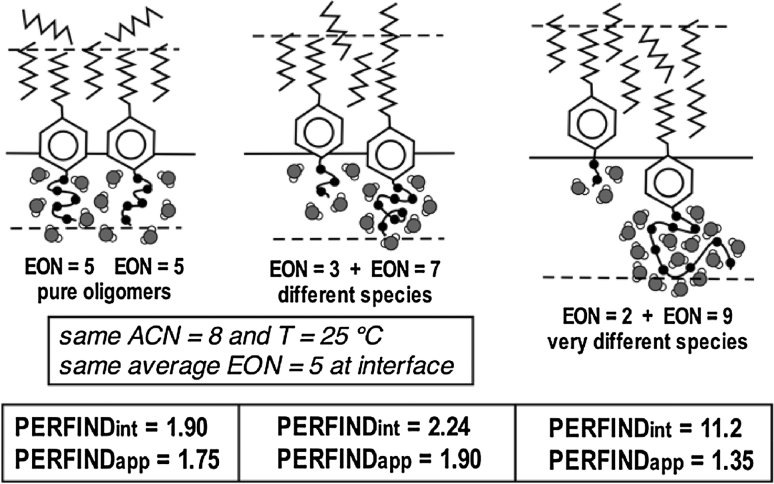
Performance at the interface and apparent in the whole system may be very different if the partitioning kicks a huge proportion of surfactant species out of the interface and microemulsion

Figure 18 referring to the mixture of two ethoxylated alkylphenols, indicates that the two SP* values and Perfind are starting to increase when the characteristics of the two surfactants deviate from each other, but that at some point of difference, the interfacial parameter (solubilization parameter SP*_int_ or Perfind_int_) continues to increase whereas the global or apparent one, viz. SP*_app_, or Perfind_app_), which is the one that corresponds to the cost, decreases when the difference is too large, e.g. here on the right hand case of Fig. 18 [58, 108].

Consequently, the principle of a mixture is still favorable to the performance at the interface even when the difference between the components becomes excessive, but it has a practical limit due to the partitioning of one or more of the species, in the present case the considerable migration of the low oligomers in the oil phase, as discussed elsewhere [43, 130, 177]. In other words, the limit of the mixture utilization is due to the departure of the species from the collective behavior at the interface to migrate into the bulk phases, and thus to be lost, as far as interfacial phenomena are concerned. This is probably the case for most lipophilic linker effects, in which a large proportion of the slightly hydrophilic compounds has partitioned into the oil phase.

This excessive partitioning problem can be made less inconvenient by introducing in the extreme hydrophilic and extreme lipophilic surfactant pair, an intermediate species that tends to reduces partitioning. Such addition of intermediates results in a more collective behavior of the mixture components and also improves the performance as resulting in the occurrence of three-phase behavior or a lower tension when the two basic extreme surfactant mixtures do not [38, 115, 131].

There is an even better approach to almost eliminate the migration of the components of the mixture because they are too hydrophilic or too lipophilic, which is to stitch them together in a single molecule whose intermediate property compels it to stay at the interface. This so-called intramolecular mixture technique consists of making new surfactant species with structures and properties similar to those of a surfactant associated with a cosurfactant or linker, but without the possibility of splitting into parts.

### Intramolecular Mixtures: Complexity and Advantages

The first surfactants with an intramolecular mixture to be studied extensively were the surfactant with a head group consisting of two parts, most often anionic and nonionic ones. The most used are the alkylethoxy sulfonates or sulfates, essentially similar in structure to the lauryl ether sulfate used in shampoos. The presence of a second polar part as 2–3 ethylene oxide groups could be thought to slightly increase the hydrophilicity and allows the possibility of having 18–20 carbon atoms in the tail without precipitation, but the main feature is a considerable increase in the salt tolerance typically form 2 to 20 % NaCl for the sulfonate and ethoxylated sulfonate counterpart [96, 98]. However, it was shown that the interaction between the ethylene oxide chain and the ionic group could result in some shielding with respect to water of the ionic part. This would decrease the hydrophilicity of the head group and a change in the degree of dissociation of the sulfate in the micelle [178]. On the other hand the presence of the ion could decrease the hydration of the ethyleneoxide groups, hence also decreasing the hydrophilicity. The improved performance may also be due to a reduced repulsion between neighboring molecules because of a variation in the distance from the interface of the ionic charge in the different species of the mixture, as illustrated in Fig. 16f, that allows lower intermolecular repulsion and thus a better density of adsorbed amphiphiles, including when mixed with conventional anionic surfactants.

Oleyl ethoxy sulfonates present a long tail with a double bond, with a salinity-ACN optimum formulation line intermediate between anionic and nonionic correlations, and with a temperature dependence (*c*_T_) like ordinary polyethoxylates but with a much lower sensitivity. The species with only three ethylene oxide groups is a particularly good performer even at high NaCl salinity or with divalent electrolytes [32, 97]. Alkyl ethoxy carboxylates with a branched tail and up to 6 ethylene oxide groups also exhibit an intermediate behavior with respect to salinity and temperature, but are very sensitive to the pH below pH 10. They exhibit good performance, e.g. above 3 units [179]. For some still unexplained reason the alkyl ethoxy carboxylic group is much more tolerant toward divalent electrolytes than the corresponding soap.

Hydroxyalkane sulfonates, which represent about 30 % of the commercial α-olefin sulfonate products, contain a small but effective second polar group, which allows using a longer tail. This tail is not branched however in most cases, and thus not as tolerant as internal olefin sulfonate corresponding species [29, 80].

The second type of intramolecular mixture deals with placing two different parts in the hydrophobic tail of the surfactant. It comes from two different research lines that have been ignoring each other over the past 30 years. As far as we know, the first started with a patent from the petroleum industry [180], proposing a structure made more salt tolerant, not by increasing the efficiency of the head group of the surfactant, but by decreasing the intolerance of the tail group to water. In di- and triblock polyethyleneoxide and polypropylene oxide copolymer surfactants, which have been used for a long time, the polypropylene oxide chain is the hydrophobic part. Of course, it is less hydrophobic than a linear or branched alkyl group, and even less than a long alkyl benzene group. In other words, it means that a polypropylene oxide chain is less likely to result in a precipitate in water or brine, and on top of it the methyl branching every three atoms reduces the hydrophobicity as seen in Part 1 of this review and also produces some steric repulsion between neighboring molecules. As a consequence, the limit in size with a polypropylene oxide hydrophobic chain is much bigger than the usual 16–18 Å of a linear alkyl group, and thus a polypropylene oxide part provides a larger tail, and a possible larger interaction, according to Winsor’s premise for improving performance. Since the polypropylene oxide chain is slightly polar, it was placed as the part of the tail which is close to the head, leaving an alkyl group at the very end of the tail. The patent included what was some hint at this time, i.e. the possibility of having a branched tail and the possibility of having a polyethylene oxide chain on the head side. The typical surfactant of this type was an alkyl propoxy ethoxy sulfonate or sulfate, with about 5 PO groups and 5 EO groups or less. However in the 1980s the crude oil price went down and the enhanced oil recovery was practically abandoned and the research in the area considerably reduced after a short experience with the first pilots. Consequently, this type of new surfactants were not extensively studied and only two exceptions containing some screening are available for surfactants with a small number of propylene oxide units and an heavily branched Guerbet tail [85, 91].

At the end of the 1980s another piece of research was carried out to solubilize polar oils like triglycerides in a microemulsion for the direct injection of oil soluble drugs into the blood for veterinary purposes. The problem to be solved was that the solubilization of polar oils, particularly triglycerides, was extremely low with conventional surfactants—both anionics and nonionics. The key was the insertion of the lipophilic linker effect inside the surfactant molecule, i.e. the introduction of a slightly polar zone in the oil bulk close to the interface. The new surfactant structure was called an extended surfactant, in which the middle part between the head and alkyl tail was a polypropylene chain with 5–15 propylene oxide groups, and eventually 1–2 ethylene oxide groups between the ionic head and the central spacer.

Extensive studies indicated the basic properties of this kind of surfactants [181]. First of all it was found that these extended surfactants were producing three-phase behavior as conventional surfactants with *n*-alkanes without any alcohol or other short cosurfactant, probably because the actual length of the polypropylene central extension varies from a molecule to the other, with a typical Gaussian distribution, as well as the branching of the polypropylene oxide chain.

It was shown that the formulation of an extended surfactant with a sulfate head changes with salinity and ACN according to the general behavior for ionic surfactants, i.e. the optimum salinity increases as the oil ACN increases with a linear lnS vs. ACN relationship [182]. As the number of propylene oxide units in the extension increases, the surfactant becomes more hydrophobic, i.e. its optimum salinity decreases, and its critical micelle concentration decreases [183]. This essentially means that the polypropylene oxide chain belongs to the tail.

What the completely new behavior was, was their capacity to have three-phase behavior with no alcohol with polar oils like mono-chain ethyl oleate, and di- or triglycerides with long fatty acids as found in edible oils. This new feature was associated with the attainment of high solubilization and low tension with polar oils, i.e. Perfind up to 2–3, instead of the usual very poor performance (Perfind below 0) for conventional surfactants [183]. The performance was found to be somehow related to the size of the polypropylene oxide extension and to the oil structure and size with different matching depending of the oil structure [182]. These properties were corroborated more recently for different structures and oil phases [18, 59, 184–197].

**Fig. 19:**
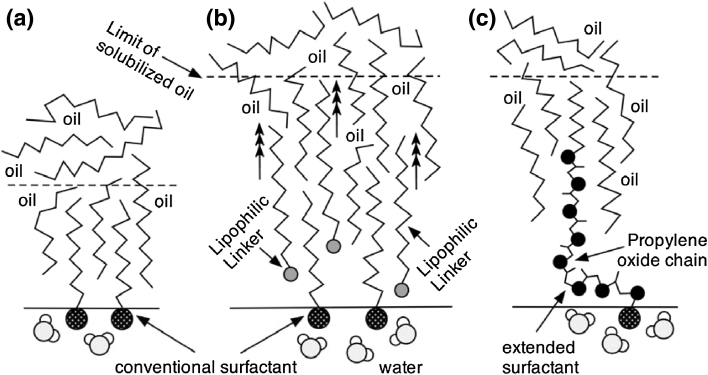
Extended surfactant (**c**) mimics the Lipophilic Linker and surfactant mixture (**b**) resulting in a much higher interaction with oil, than in the case having only a conventional surfactant (**a**)

As for the case of the lipophilic linker effect [149], the performance seems to increase when both the tail end alkyl chain and intermediate propylene oxide extension increase as seen in the Fig. 19b, c illustrations [59]. Because of its hydrophobic character, the polypropylene central part is essentially in the oil phase, but there is some evidence that it is bent to some extent to be close to the interface, as indicated in Fig. 19c. In effect, the behavior as the temperature increases was unexpectedly found to be similar to the case of the polyethoxylated nonionics, although weaker, i.e. they become more hydrophobic when the temperature increases [194]. This cannot be explained except by the dehydration of some polypropylene oxide units which have to be close to the interface. Since there is essentially no change when there are more than 2–3 polypropylene oxide units, the bending is assumed to be limited to these first groups. This results in an approximate 10-Å considerable lateral steric disorder and thus a perfect explanation for the welcome characteristics of the absence of structure with no alcohol. This is probably a contribution to a more complex effect due to details in the tail structure as recently discussed [19].

An unusual feature of this kind of surfactant is that the performance has been found to improve in some cases when both, the salinity and ACN, increases, which is an exception to the usual trends, which may be important in practice, particularly in enhanced oil recovery [59, 192]. Another favorable hint is that this kind of surfactant is probably more likely to solubilize the polar oil species found close to the interface in crude oils like asphaltenes and resins, and consequently would exhibit a better performance than common conventional surfactants.

A variety of extended surfactants have been proposed, not only by changing the tail branching [91, 188, 189, 193, 198, 199] but also the head group with sugar or carboxylate, or phosphate combinations. Several applications have been proposed for these surfactants particularly because of their capacity to solubilize natural triglyceride oils in pharmaceutical vehicles, detergency [200], agroindustrial oil extraction [201], crude oil demulsification [202] as well as some others mentioned in a previous review on solubilization [46].

The main justification of the success of these structures is that they are able to increase the thickness of the interfacial layer considerably, which includes at the same time the features provided by Winsor’s premise but with no precipitation nor liquid crystal, and by the intermolecular mixture benefit but no partitioning. Additionally the extended surfactant in its complete details provides the combination of a conventional surfactant with lipophilic and hydrophilic linkers, but no partitioning. The interfacial layer thus exhibits a continuous variation in the polarity from oil to water, with a thick transition zone which is likely to be associated to a better compatibility between oil and water and thus a better performance as discussed in previous years [59, 203, 204].

In a recent report, an intermediate spacer made of 7 propylene oxide units and 7 ethylene oxide units, has been made either in two sequential blocks or in a random chain. The extended surfactant performed when the two alkoxides were in sequence, i.e. when there was a continuous progression from lipophilic to hydrophilic [193].

This approach has been recently corroborated by the report of a better performance if the intermediate spacer contains the sequence polybutylene oxide-polypropylene oxide-polyethyleneoxide [115]. In such a sequential arrangement, the butylene oxide that has been used in triblock copolymer surfactants [205], provides an even less hydrophilic tail portion than polypropylene oxide.

It is worth noting that the first extended surfactants for petroleum recovery contained about 5 propylene oxide units, while the ones used with polar oils solubilization had about 15 units, and recently proposed ones could have many more. In other words the size of the intermediate spacer could represent more than 90 % of the extended surfactant length, and the structure would not really contain a head and a tail, but a long sequence of segments from very lipophilic to very hydrophilic properties.

Since the alkoxide addition is a random reaction, the actual number of units of propylene oxide (and eventually ethylene or butylene oxide), would vary from one molecule to the next and thus provides an extra reason to avoid a too rigid structure. This is likely to increase the tolerance to precipitation effects and even if it is not the case, it could be added in a small enough amount in a mixture, to be quite dispersed at the interface as the diblock amphiphilic copolymers [116] mentioned previously. It is worth remarking that these extra-big copolymeric surfactants were recently called amphiphilic linkers [206], to insist on the mechanism of this exceptional performance boosting role which is probably due to the right compromise between an increased global rigidity of the interfacial layer structure with all parts stitched together, but with an intrinsic flexibility for the non uniformity of the molecules, particularly in the spacer part.

Recently, extra large extended surfactants species have been proposed with molecular weight in the 2000–3000 Da range [207, 208], i.e. much larger that ordinary surfactants, not yet as large as amphiphilic linker copolymer [116, 117], but probably less likely to precipitate at low concentration. This trend obviously follows the previous success of amphiphilic copolymers and there is probably some margin to increase the size even more, and thus the performance, in particular for the introduction of a larger but less hydrophobic tail than with the copolymer.

This gathering of different advantages in the extended surfactant structure is probably the reason why extended surfactant have been found to be almost compulsory in mixtures suggested in the recently proposed formulas in enhanced oil recovery, either as some general advice [209–211] or somehow presented as a magic feature as done in many symposium talks [115].

**Fig. 20:**
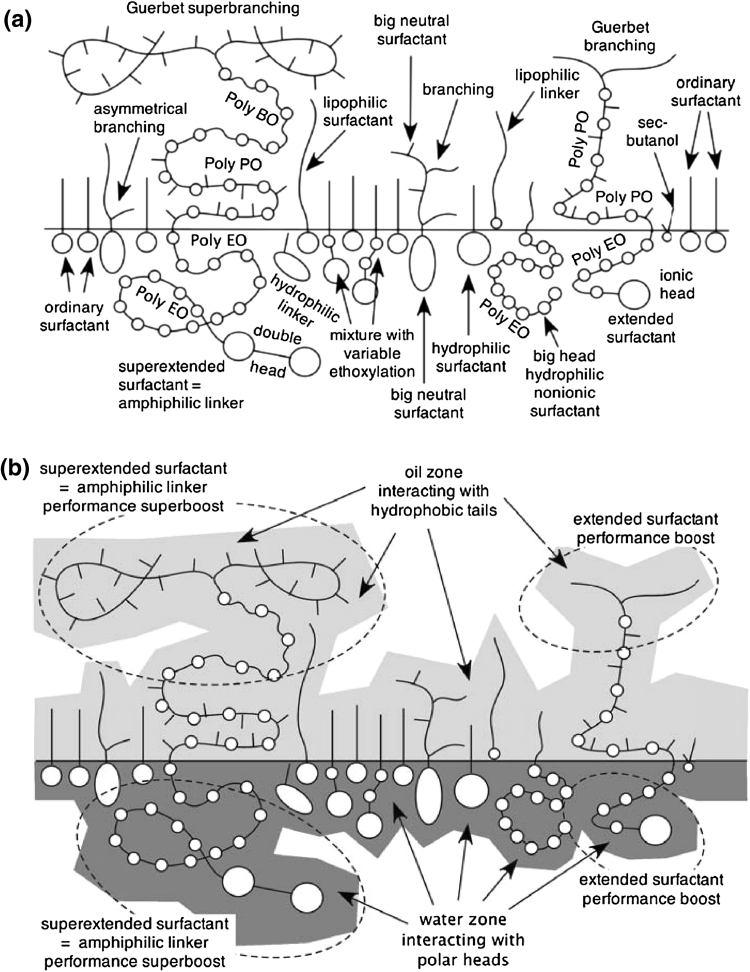
Inventive schematic gathering of the different cases of current know-how on how to increase performance in enhanced oil recovery. **a** Different molecules in the interfacial mixture; **b** oil and water zones interacting with these molecules

Figure 20 is an inventive schematic of what could be the very best optimum formulation of the interface. It gathers the many contributions susceptible to improve low tension performance in enhanced oil recovery, all together as may be the best way to take advantage of the current knowhow. All these species have been proposed and tested, with the exception of the so-called super-extended surfactant, which will probably appear soon as a way to approach the multiblock amphiphilic linker size.

Figure 20a indicates the different molecules appearing in this guessed magic mixture formulation, while Fig. 20b indicate the zones of oil and water which interact with the amphiphiles at the interface, and are thus solubilized in a microemulsion. This is directly related to performance, since according to Huh’s relationship, the higher the solubilization zone, the lower the tension.

It is worth noting as the final remark that it has been shown that extended surfactants mix well with other surfactants, but with a particular and welcomed characteristic. There is some evidence that the mixing rules with conventional surfactants are followed as far as the optimum formulation SAD/HLD is concerned as will be discussed in the third part of this review. However, it has been shown a long time ago that the predicted performance effect has to be handled separately from the formulation [212], probably because it depends on different variables.

With edible triglyceride oils, in which case the conventional surfactants are not efficient, it was found that the performance of the mixture mainly depends on the contribution of the extended surfactant. This might also depend on the oil structure, since this early finding [212] was recently corroborated as being more general by a study with pure alcohol ethoxylates [60] that better solubilize alkanes than triglycerides even at the same EACN. If for hydrocarbons both type of surfactants are likely to contribute to the performance, a large extended surfactant probably has a dominant role as an amphiphilic linker even if it is in a much smaller amount, and this is probably the main motivation to incorporate it into a formulation for enhanced oil recovery.

This is no wonder if it is remembered that some of the performance contribution depends on what happens exactly at the interface according to Winsor’s approach, while other performance effects do not, but depend on what happens in the bulk phases as in the case of the linker effects.

## Conclusions

As a conclusion to this second part of the review on applications for enhanced oil recovery, it may be said that many different influences are involved and that the current know-how is still extremely complex and not clear enough for finding a unique straightforward path to an optimum compromise between a higher interfacial rigidity and enough flexibility to avoid organized structures occurring.

The attainment of an optimum formulation will have to take into account what has been discussed in the two first parts of this review, with some other restrictions concerning phenomena of importance like adsorption and effects susceptible to change the enhanced oil recovery conditions.

In any case, the current practical problem is that after reading this review, it is easy to draw one or various other surfactant molecules with some probability of providing the right combination of properties as seen in Fig. 20. However, the most likely occurrence is that such magic species are not all commercially available and it might not be easy to produce them at a competitive price, particularly the extended surfactant species. In the 1970s many petroleum sulfonates were available to test, but it is not the case now. There are only a very few companies that produce relatively few surfactants with many characteristics reported in this review, and since they cannot offer scores of different products to cover all the particular cases, the choice is limited to what is available. It is quite likely that, in the near future, a few more products will be proposed so that a reasonable choice will be available to cover most of the issues discussed. This might help the formulator to handle the complex features to prepare high performance mixtures, as will be discussed in the third part of this review.

On the other hand, the simple change from a petroleum field to another will result in a change in crude, i.e. oil EACN, connate water salinity and reservoir temperature, which might change a lot of things, particularly the best compromise and thus the best enhanced oil recovery formulation for each case. Consequently a new best formulation will have to be guessed for every field. Understanding the phenomena and gaining experience on using the current know-how in screening techniques is obviously the desirable and probably compulsory expertise for the formulator.
